# Supplementary material for: GS9 acts as a transcriptional activator to regulate rice grain shape and appearance quality
Source: Nat Commun. 2018 Mar 27;9:1240. doi: 10.1038/s41467-018-03616-y (PMC5869696; doi:10.1038/s41467-018-03616-y)
Supplement: Supplementary file 1 — Supplementary Information(DOCX 10228 kb) [file 41467_2018_3616_MOESM1_ESM.docx]

***GS9* acts as a transcriptional activator to regulate rice grain shape and appearance quality**

Zhao *et al.*


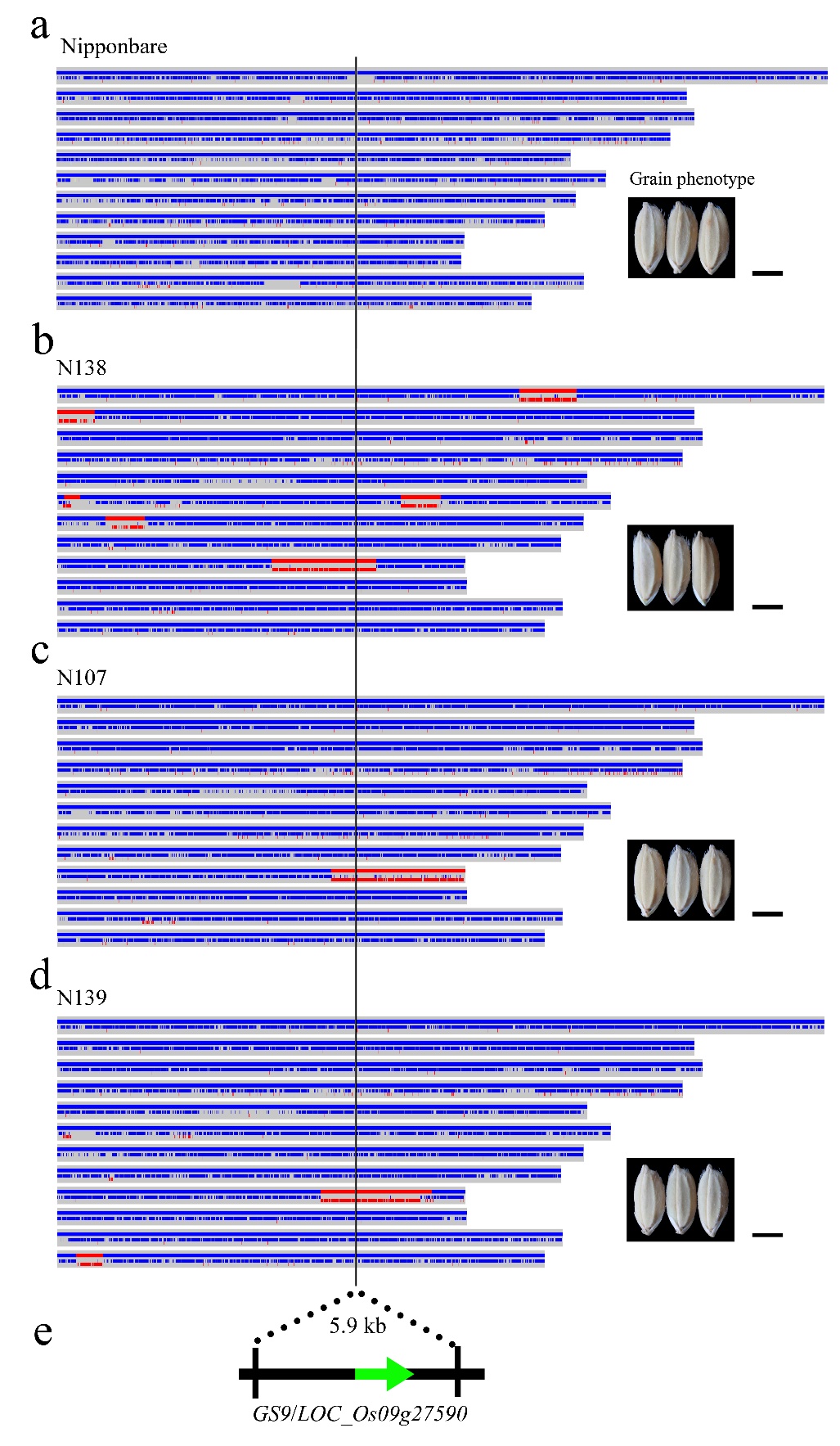


**Supplementary Figure 1.** **The genotype and grain morphology of three chromosome segment substitutional lines and their recipient Nipponbare.** (a-d) The genotype of Nipponbare and its three chromosome segment substitutional lines (CSSLs), N138, N107, and N139. The red and blue bars indicate the chromosome fragments from donor and recipient revealed by reduced genome re-sequencing approach, respectively. N138 and N139 lines are derived from the *japonica* Nipponbare (recipient) and *indica* Qingluzan11 (donor), while N107 is from Nipponbare (recipient) and another *indica* 9311 (donor). The mature grain morphology was shown in right (scale bar, 3 mm), and only the N138 line conferred a more slender grain phenotype than Nipponbare and other two CSSLs. (e) A 5.9-kb delimited region was located in the introgressed fragment on chromosome 9 and contained only one open reading frame, *LOC_Os09g27590*, which is thought to be responsible for grain phenotype. The black vertical line indicates the delimited mapping region.


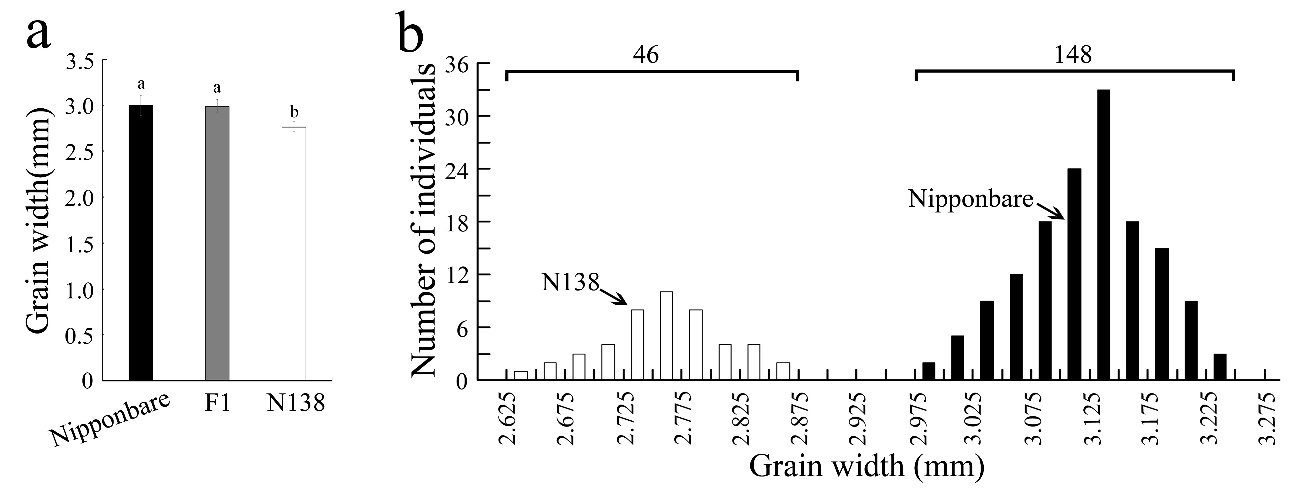


**Supplementary Figure 2. Genetic analysis of *GS9* locus.** (a) The width of mature grains from Nipponbare, N138 and their F_1_ hybrid. Data are given as means ± SD, n=15. Different letters represent a significant difference (*P* < 0.01, one-way ANOVA). (b) Distribution of mature grain width among 194 F_2_ individuals derived from the cross between Nipponbare and N138. The grain width of N138 and Nipponbare were specially indicated by arrow. The white and black columns represented the narrow or normal grain phenotype as those of N138 line and Nipponbare, respectively. 148 and 46 are the number of F_2_ individuals with normal and narrow grains, respectively. Numbers of grains based on phenotype were used for Chi-Square test. *P* = 0.678 > 0.05, not significant.


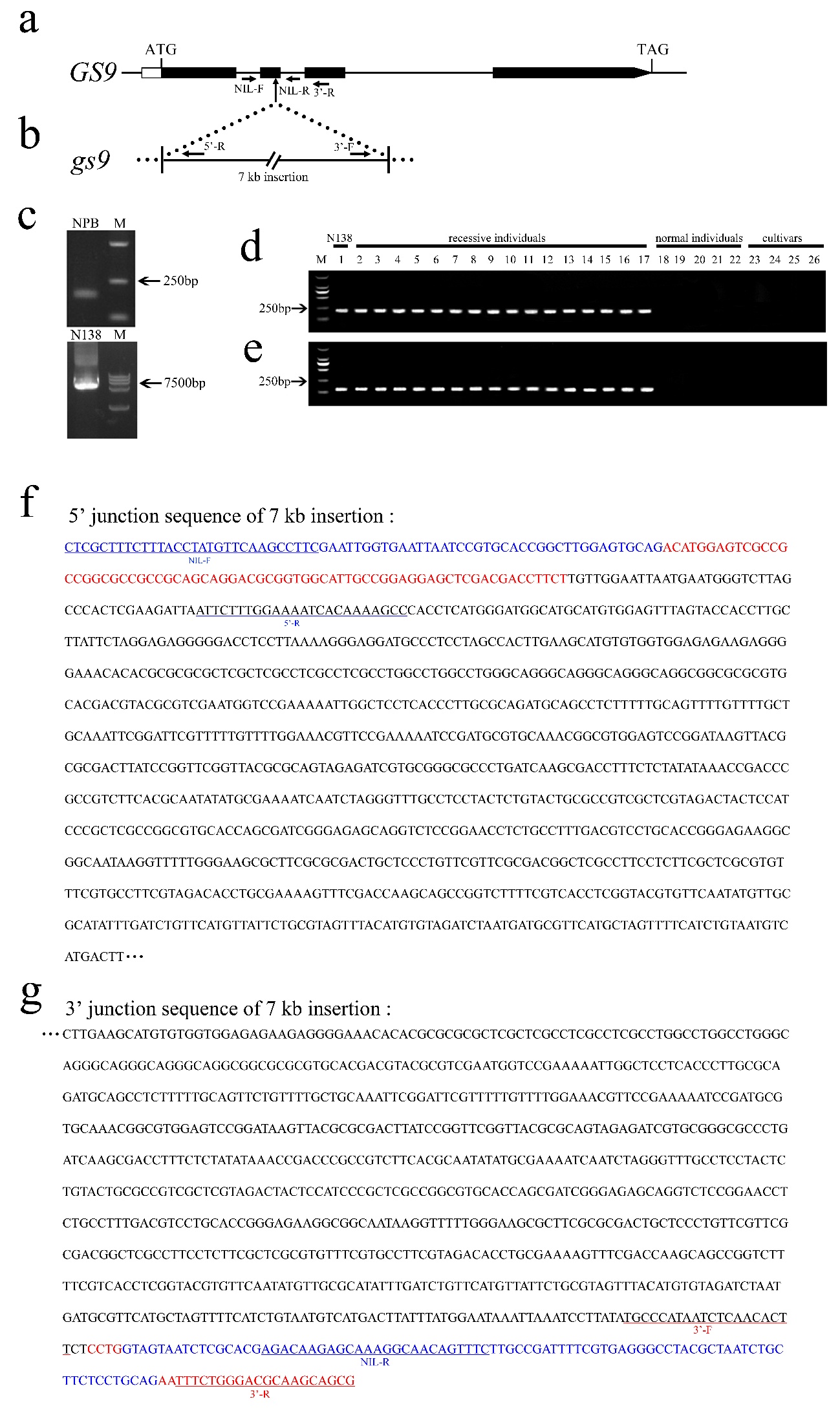


**Supplementary Figure 3.** **Identification of the 7-kb insertion in *gs9* allele of N138 line.** (a, b) The structure of *GS9* and *gs9* alleles from Nipponbare and N138, respectively. The horizontal arrows show the sites of primers for junction and co-segregation analyses, and the vertical arrow indicates the site of 7-kb insertion in exon 2 of *gs9* allele. (c) PCR amplification by using the primers NIL-F and NIL-R located around the 7-kb insertion site. A large fragment (~7-kb) was amplified from the genome of N138 line with *gs9* allele, while only a small product was amplified from wild type Nipponbare (NPB). (d-e) Co-segregation analysis between the grain shape and 7-kb insertion within the F_2_ population from N138 and Nipponbare. The primers NIL-F/5’-R (d) and 3’-F/3’-R (e) were used to amplify the right or left junction of 7-kb insertion, respectively. Lane 1, N138 line; Lanes 2-17, F_2_ recessive individuals with slender grain shape as N138; Lanes 18-22, F_2_ normal individuals with wide grain shape as Nipponbare; Lanes 23-26, four rice cultivars, Qingluzan11, 9311, Nipponbare and Lemont, respectively. The data showed that there were PCR products from rice samples on lanes 1-21 with slender grain phenotype and *gs9* allele due to the 7-kb insertion, while no PCR products from samples on lanes 22-26 with normal grain shape and *GS9* allele. Therefore, the insert fragment co-segregated with the mutant grain shape. (f, g) The 5’- (f) and (g) 3’- junction sequences of the 7-kb insertion from the genome of N138 line with *gs9* allele. Blue and red letters represent the intron and exon regions of *GS9* gene, and black letters represent the insertion fragment. The locations of primers shown in panels (a) and (b) are labeled with horizontal lines.

**
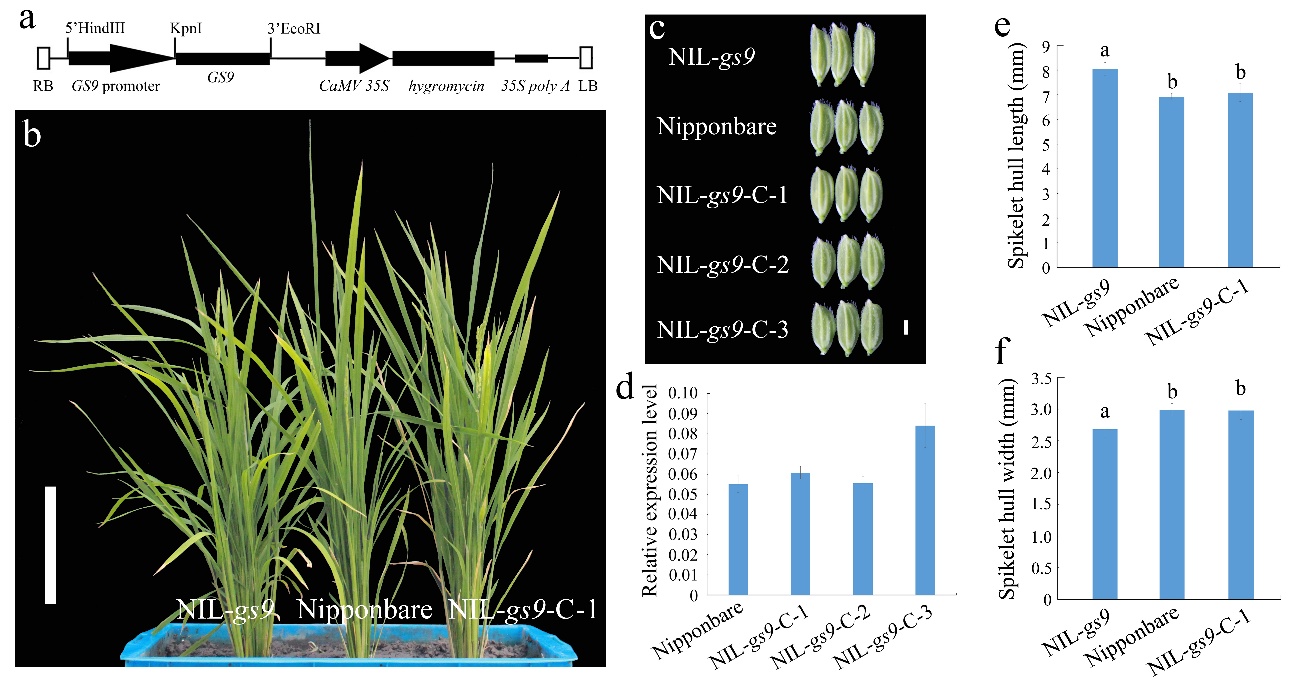
**

**Supplementary Figure 4. Complementary test of *GS9*.** (a) The T-DNA region of complementary vector pGS9::GS9. The full-length CDS of *GS9* gene was driven by its 2.3-kb native promoter, and both were cloned from Nipponbare. The hygromycin resistance gene, controlled by the *CaMV 35S* promoter, is as the selectable marker during rice transformation. (b) Plant morphology before flowering. NIL-*gs9*, the near isogenic line carrying *gs9* allele in Nipponbare background; NIL-*gs9*-C, T_0_ transgenic plant derived from NIL-*gs9* with the complementary vector pGS9::GS9. Scale bar, 20 cm. (c) The phenotype of spikelet hulls just before flowering. Scale bar, 2 mm. (d) Transcriptional expression of *GS9* in young panicles by real-time qRT-PCR. The expression level of complementary plants resembled that of wild-type Nipponbare. Data are given as means ± SD, n=3. (e-f) The length (e) and width (f) of spikelet hull just before flowering. Data are given as means ± SD, n=15. Different letters represent a significant difference (*P* < 0.01, one-way ANOVA).


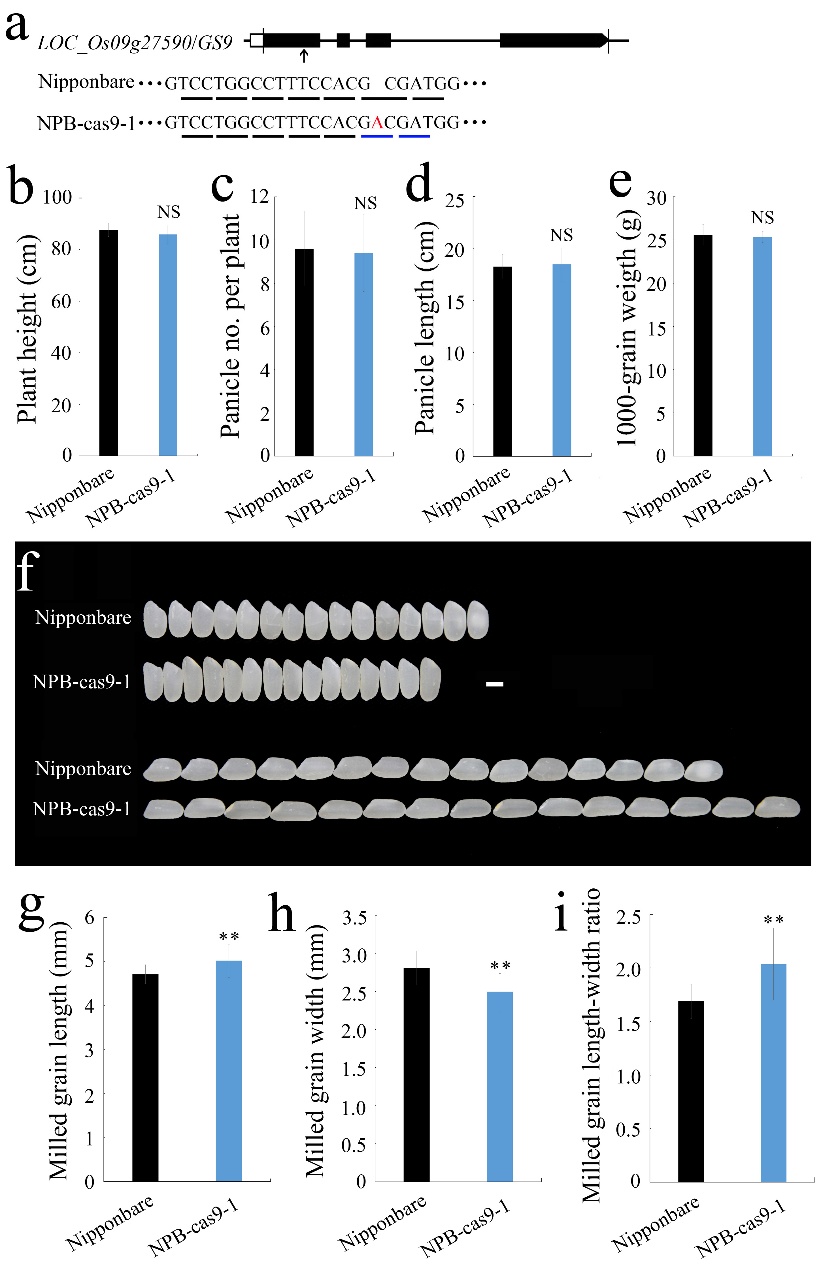


**Supplementary Figure 5.** **The *gs9* loss-of-function mutant generated by CRISPR/Cas9 genome editing system in Nipponbare background.** (a) The edited target sequences on *GS9* gene. The vertical arrow indicates the site for editing. NPB-cas9-1, the *gs9* loss-of-function mutant resulted from a single base insertion into the *GS9* gene of Nipponbare. (b-e) Comparison of the major agronomic traits between NPB-cas9-1 and its wild type Nipponbare. Data are given as means ± SD, n=10 in (b-d) or 3 in (e). NS, not significant difference (*t*-test). (f) Comparison of the shape of milled rice between Nipponbare and NPB-cas9-1. Scale bar, 2 mm. (g-i) The length, width, and the length-width ratio of milled rice from Nipponbare and NPB-cas9-1. Data are given as means ± SD, n=170. **, significant difference (*P* < 0.01, *t*-test).


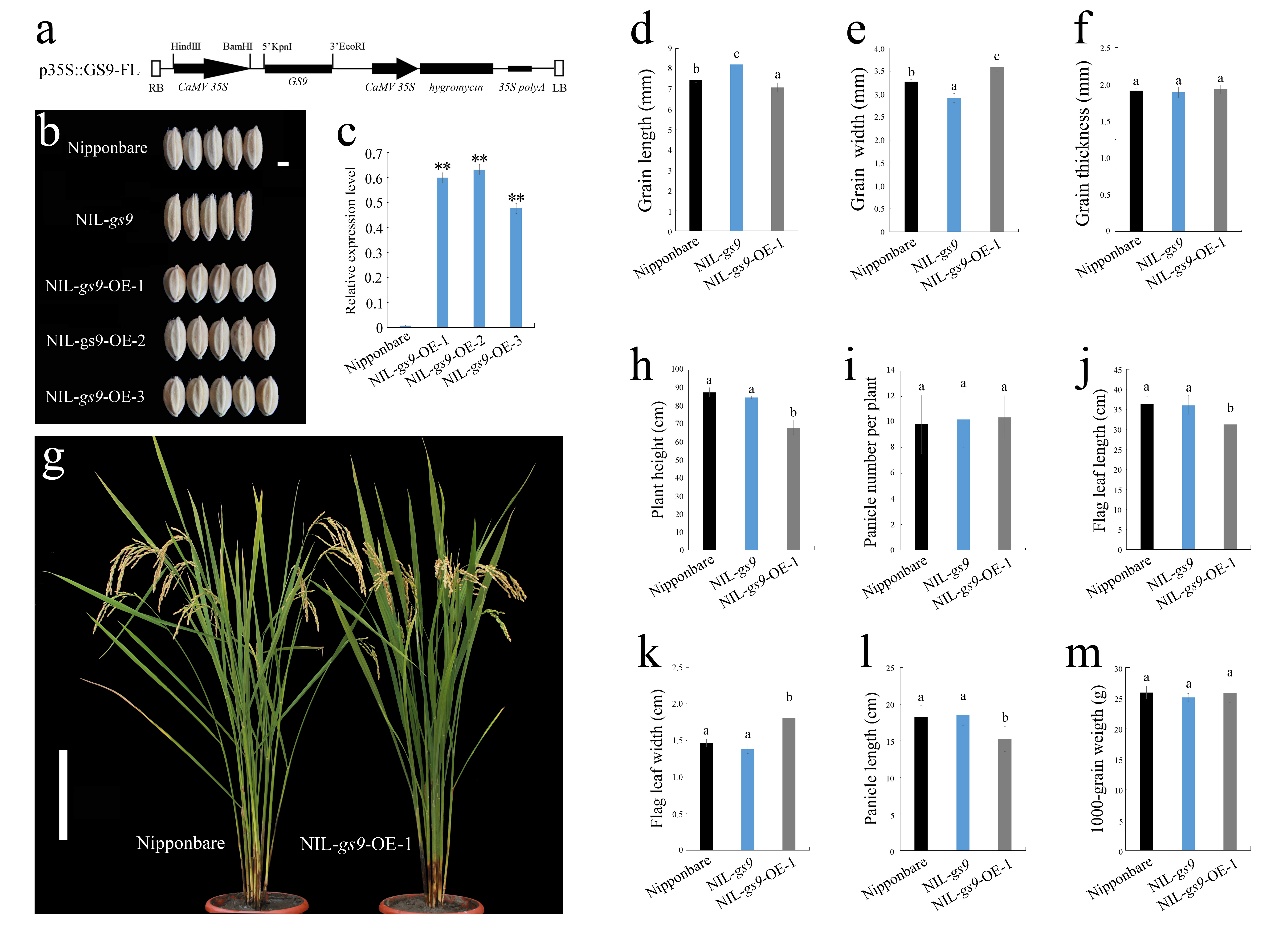


**Supplementary Figure 6.** **The phenotype of *GS9* overexpression plants in NIL-*gs9* background.** (a) T-DNA region of overexpression vector p35S::GS9-FL. The full-length CDS of *GS9* gene was driven by the *CaMV 35S* promoter. (b) The morphology of mature grains. NIL-*gs9*, the near-isogenic line carrying *gs9* allele from N138 in Nipponbare background. NIL-*gs9*-OE, *GS9* overexpression lines derived from NIL-*gs9*. Scale bar, 2 mm. (c) Transcriptional expression of *GS9* in young panicles by real-time qRT-PCR. Data are given as means ± SD, with three biological replicates. **, significant difference compared with Nipponbare (*P* < 0.01, *t*-test). (d-f) Comparison of the length, width and thickness of mature grains among NIL-*gs9*-OE, NIL-*gs9* and Nipponbare. Data are given as means ± SD, n=30. Different letters mean significant difference (*P* < 0.01, one-way ANOVA). (g) The plant morphology after maturation. Scale bar, 20 cm. (h-m) Comparison of the major agronomic traits among NIL-*gs9*-OE, NIL-*gs9* and Nipponbare. Data are given as means ± SD. n=10 in (h-l) or 3 in (m). Different letters mean significant difference (*P* < 0.01, one-way ANOVA).


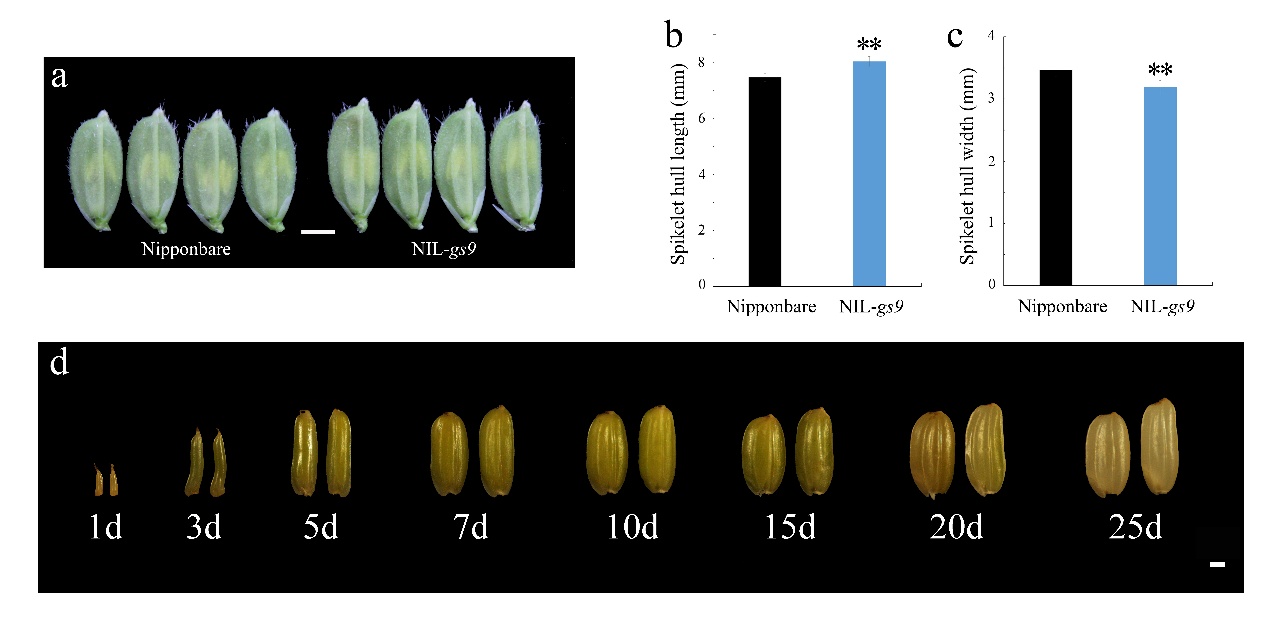


**Supplementary Figure 7.** **Morphology of spikelet hulls and developing caryopsis from Nipponbare and NIL-*gs9*.** (a-c) The morphology (a) and length (b) and width (c) of spikelet hulls just before flowering. Scale bar, 2 mm. Data are given as means ± SD, n=30. **, significant difference (*P* < 0.01, *t*-test). (d) Comparison of the shape of developing caryopsis at indicated days after fertilization between Nipponbare (left) and NIL-*gs9* (right). Scale bar, 1 mm.


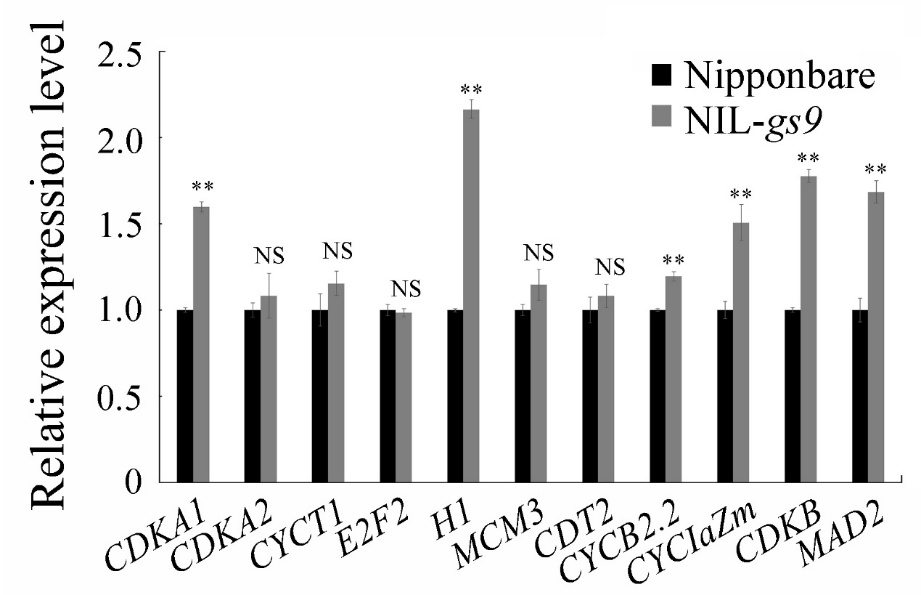


**Supplementary Figure 8. The relative expression of 11 cell cycle related genes in young panicles of NIL-*gs9* and Nipponbare.** *CDKA1*, *LOC_Os03g02680*; *CDKA2*, *LOC_Os02g03060*; *CYCT1*, *LOC_Os02g24190*; *E2F2*, *LOC_Os12g06200*; *H1*, *LOC_Os04g18090*; *MCM3*, *LOC_Os05g39850*; *CDT2*, *LOC_Os03g49200*; *CYCB2.2*, *LOC_Os06g51110*; *CYCIaZm*, *LOC_Os01g59120*; *CDKB*, *LOC_Os08g40170*; *MAD2*, *LOC_Os04g40940*. *Actin* was used as the internal control. Data are given as mean ± SD, n=3. **, significant difference (*P* < 0.01, *t*-test). NS, not significant.


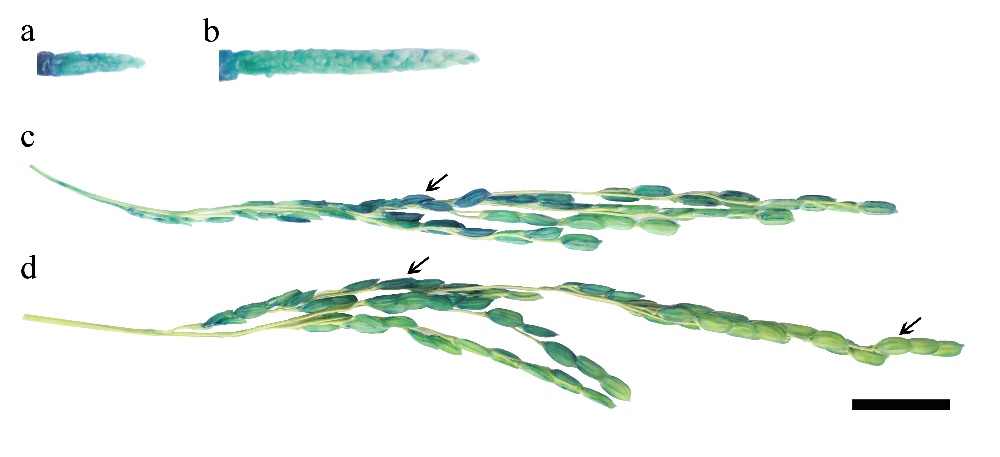


**Supplementary Figure 9.** **Histochemical assay of GUS activity in panicles at different stages of transgenic rice plants.** (a-b) Inflorescence at different developing stages. (c-d) Panicles at different developing stages. Arrows indicate strong or weak GUS activity in the spikelet hulls. The GUS reporter gene was driven by the 2.3-kb *GS9* native promoter. Strong GUS expression was observed in the spikelet hulls during panicle development. Scale bar, 1 cm.


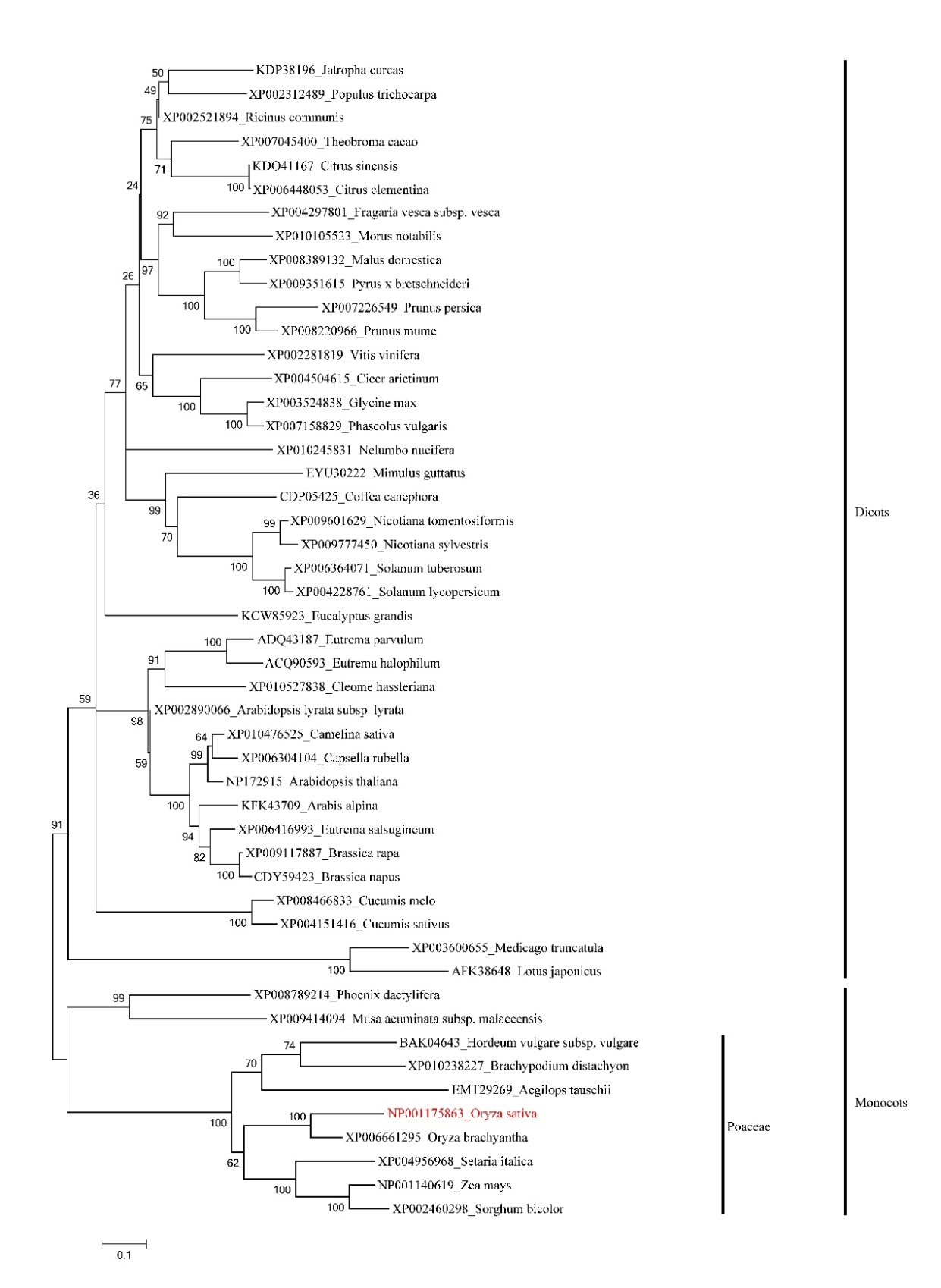


**Supplementary Figure 10.** **Phylogenetic tree analysis of GS9 and GS9-like proteins.** Dicots and monocots were classified into two subgroups. Full-length amino acid sequences were obtained from the NCBI, and the corresponding accession numbers were shown in front of the species name in a simplified form. The red one indicates the predicted GS9 protein in *Oryza sativa*.


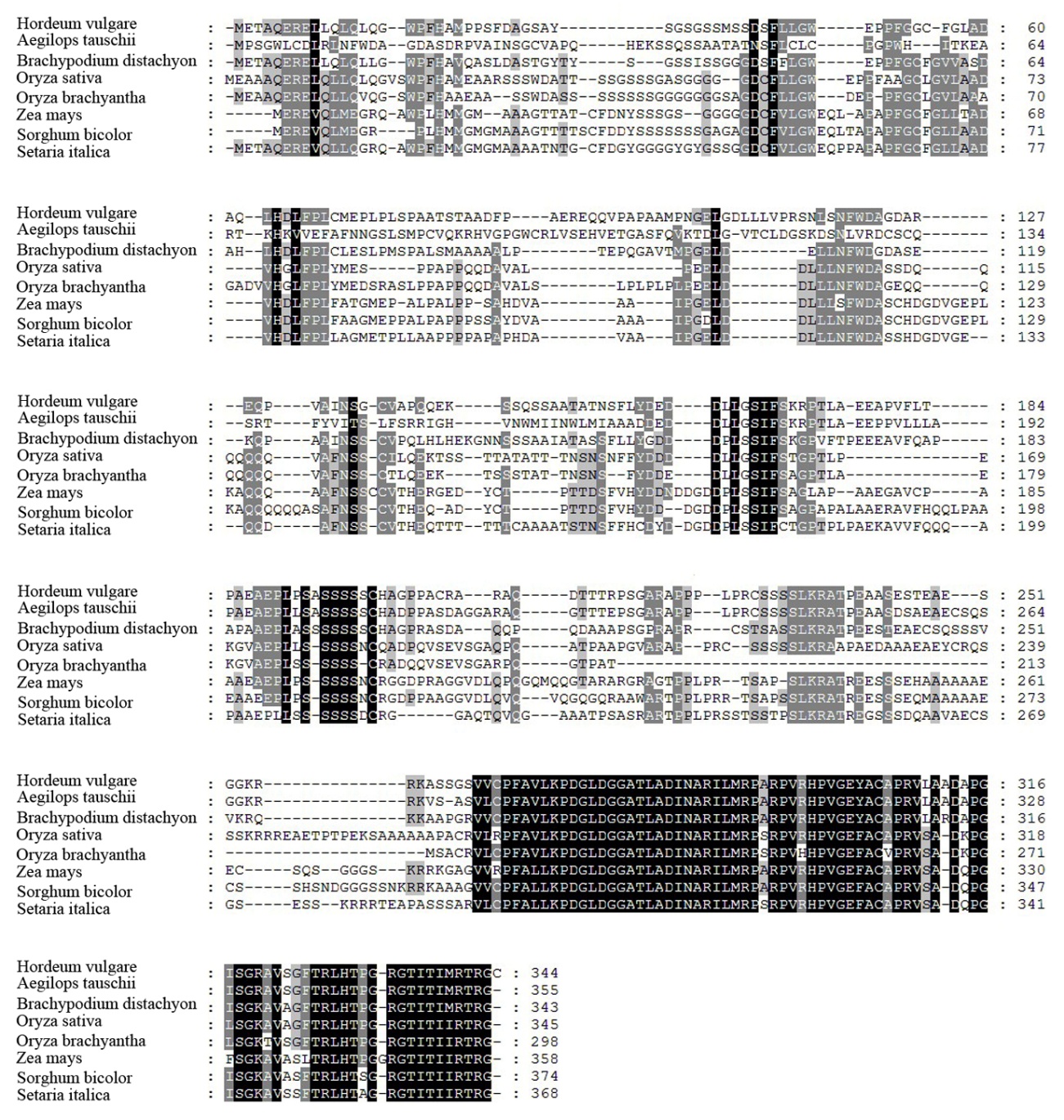


**Supplementary Figure 11.** **Sequence alignment of GS9 and GS9-like predicted proteins among Poaceae.** *Oryza sativa* represents the full-length amino acid sequence of GS9/LOC_Os09g27590 in rice. Full-length amino acid sequences of other certain species were all obtained from the NCBI website, and their corresponding accession numbers were as same as shown in Supplementary Figure 10.


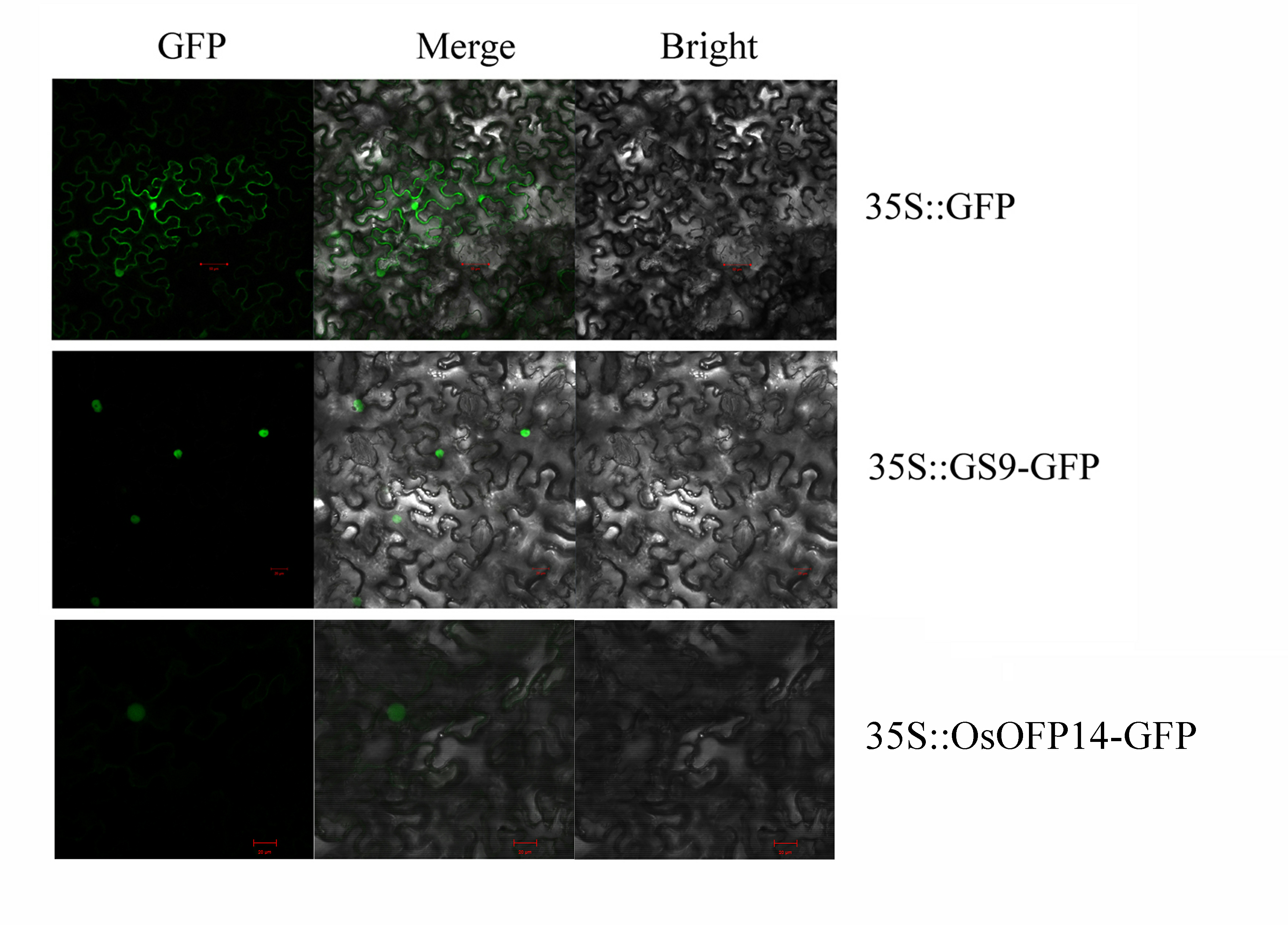


**Supplementary Figure 12.** **Subcellular localization of GS9 and OsOFP14 in tobacco leaf cells.** The 35S::GS9-GFP and 35S::OsOFP14-GFP constructs contained full-length cDNA of *GS9* or *OsOFP14* in-frame fusion with *GFP* coding region and driven by the *CaMV 35S* promoter, respectively. The constructs and their corresponding empty vector 35S::GFP were transformed into tobacco leaves via *Agrobacterium*, respectively. Scale bars, 50 μm in empty vector 35S::GFP and 20 μm in the fusion constructs.


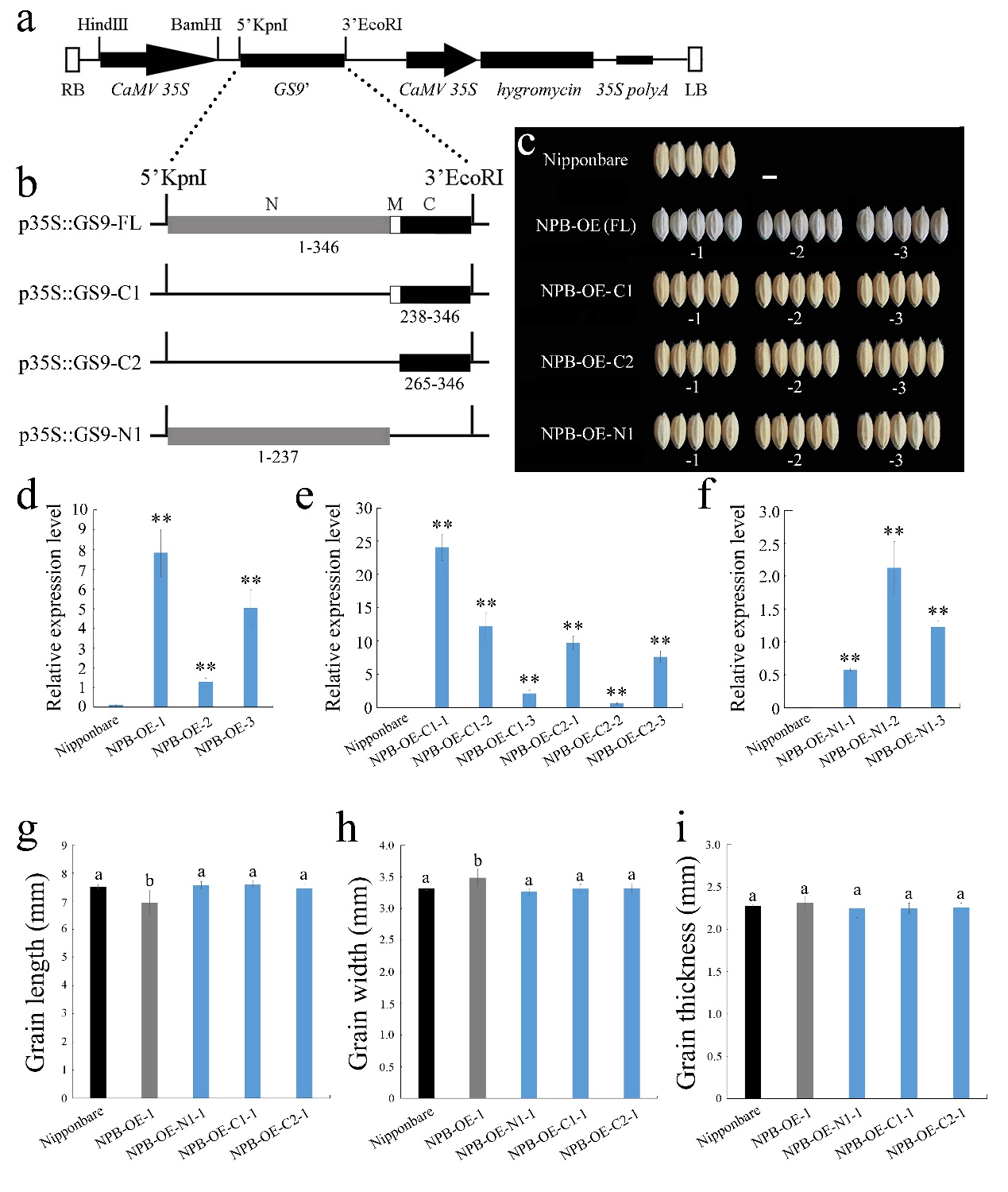


**Supplementary Figure 13.** **Overexpression of different GS9 truncations in rice.** (a-b) T-DNA region of overexpression vectors with full-length or truncations of GS9. p35S::GS9-FL contains full-length *GS9* CDS (encoding 1-346 residues), and p35S::GS9-C1, p35S::GS9-C2 or p35S::GS9-N1 only contain the regions encoding the 238-346 or 265-346 residues of C-terminal, or 1-237 residues of N-terminal of GS9, respectively. All were driven by the *CaMV 35S* promoter. GS9’ represents the full-length and truncations of GS9. N, N-terminal of GS9; M, middle part of GS9; C, C-terminal of GS9. (c) Morphology of mature grains from overexpression plants. NPB-OE-FL, NPB-OE-C1, NPB-OE-C2 and NPB-OE-N1 represented the transgenic plants derived from Nipponbare with the corresponding vectors as shown in panel (b), respectively. Three independent transgenic lines were shown for each type of transformation. Scale bar, 3mm. (d-f) Transcriptional expression of *GS9* or introduced truncated *GS9* in young panicles by real-time qRT-PCR. Data are given as means ± SD, with three biological replicates. **, significant difference compared with Nipponbare (*P* < 0.01, *t*-test). Levels increased significantly compared with Nipponbare. (g-i) Comparison of grain shape among different overexpression transgenic lines. Data are given as means ± SD, n=30. Different letters mean significant difference (*P* < 0.01, one-way ANOVA). The truncations of GS9 did not affect grain shape.


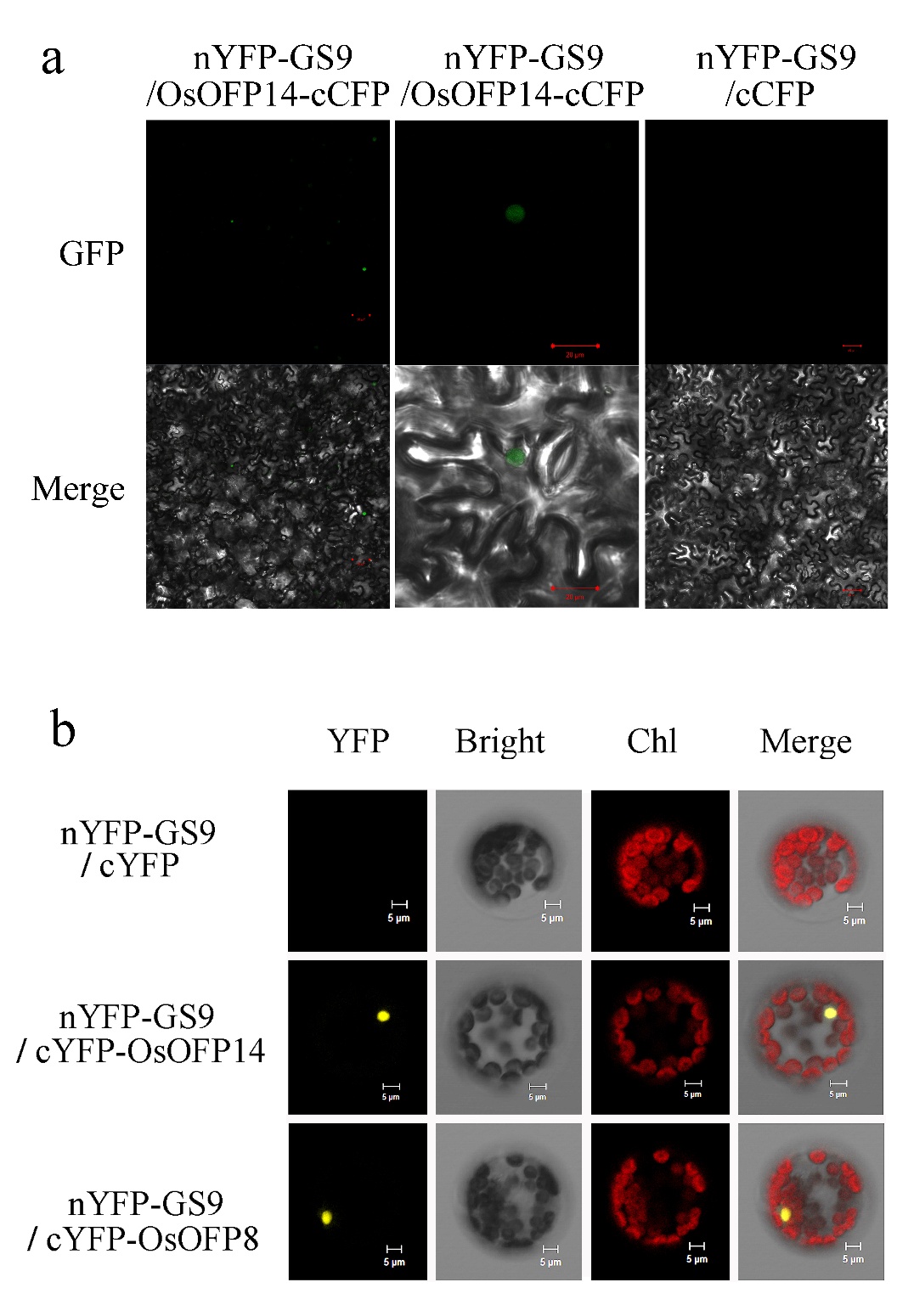


**Supplementary Figure 14. Confirmation of the interaction between GS9 and OsOFP14, OsOFP8 using BiFC system.** (a) the interaction between GS9 and OsOFP14 in tobacco leaves epidermal cells. Scale bar, 50 μm in left; 20 μm in middle; Scale bar, 50 μm in right. (b) the interaction between GS9 and OsOFP14, OsOFP8 in Arabidopsis protoplasts. nYFP and cYFP, N-terminal and C-terminal of YFP, respectively. Chl, Chlorophyll. Scale bar, 5μm.


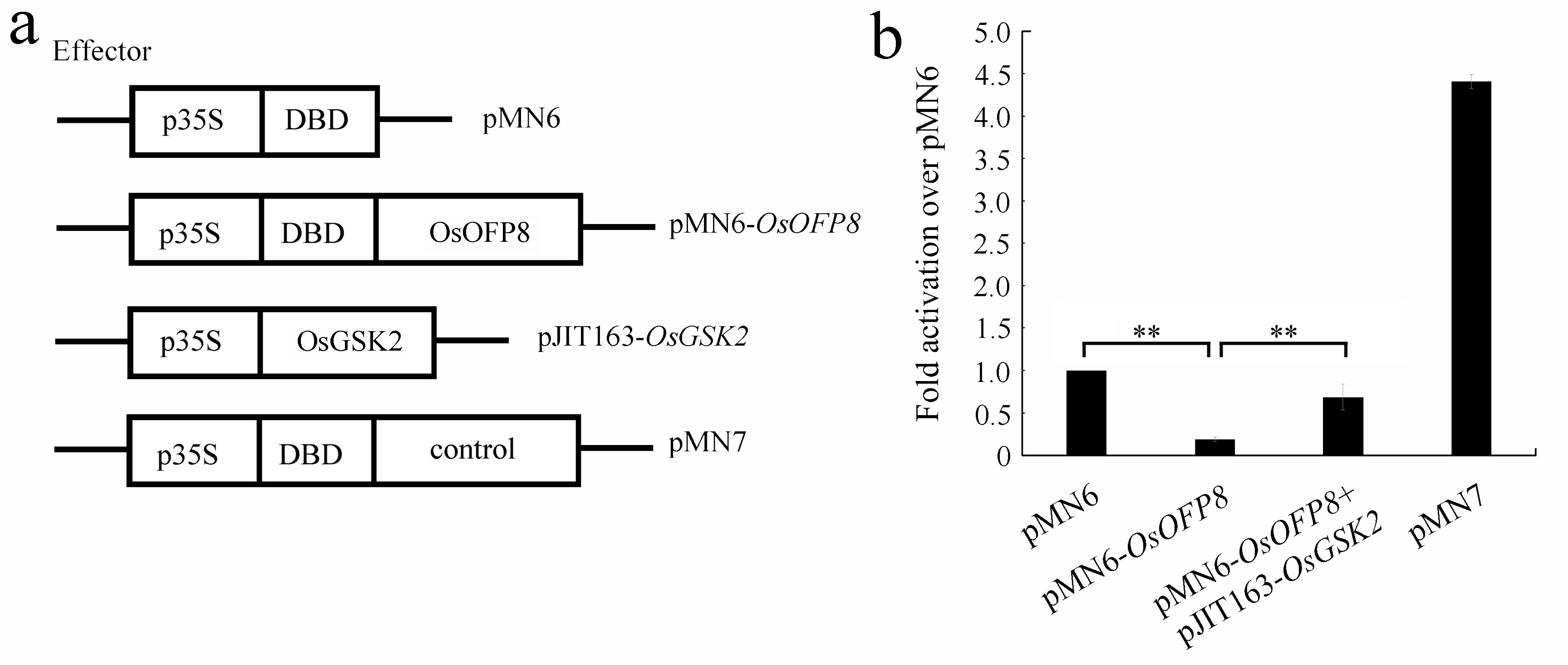
**Supplementary Figure 15.** **Transcription activity assay of *OsOFP8* and *OsGSK2* using the pMN6 system.** (a) The main structure of effector vectors. (b) The transcription activity in *Arabidopsis* protoplasts by co-transformation of different effector vector(s) with the reporter plasmid pGIL and internal control pRIL as same with that in **Figure 5c**. Data are given as means ± SD, with three biological replicates. **, significant difference (*P* < 0.01, *t*-test).


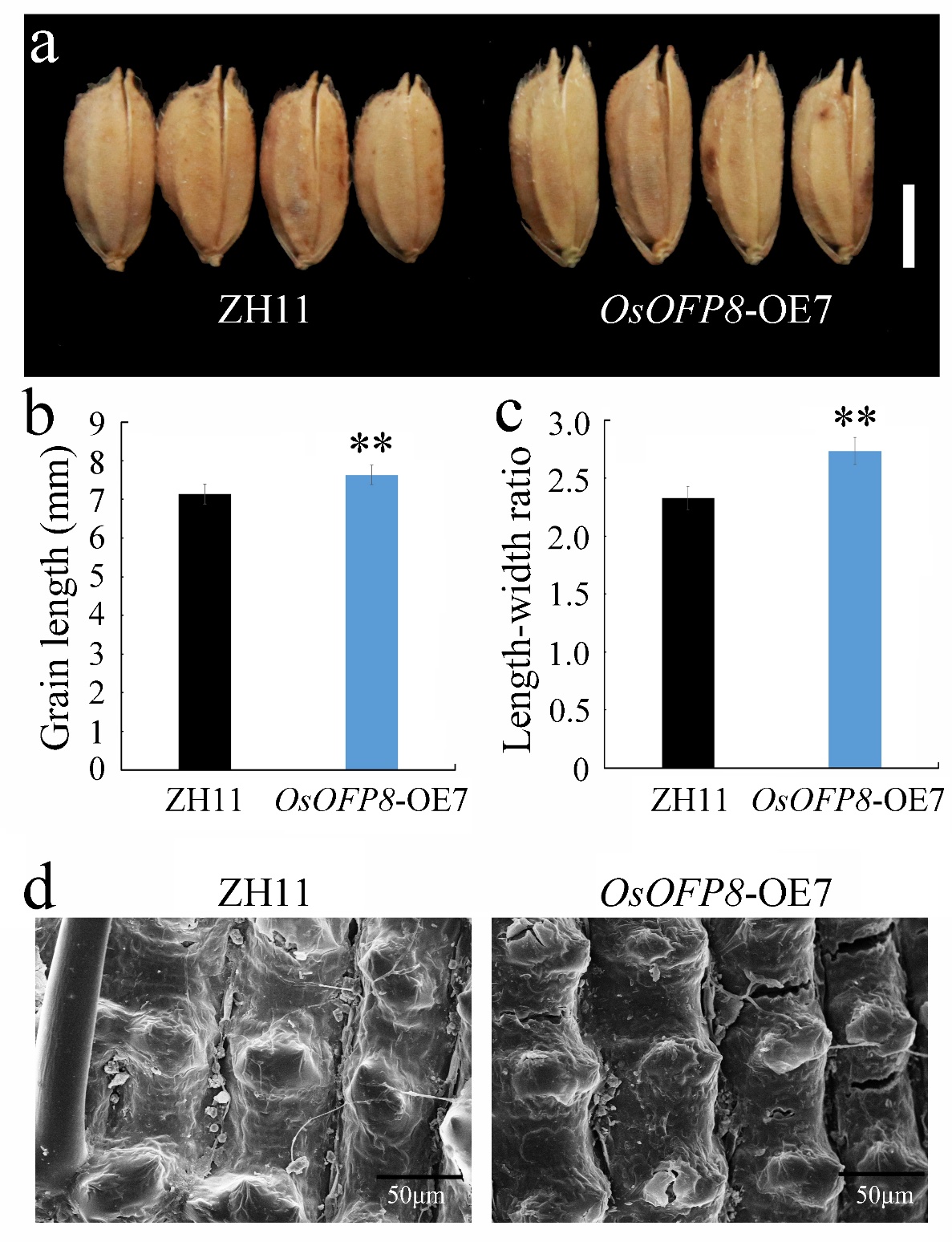


**Supplementary Figure 16.** **Comparison of the mature grains between *OsOFP8*-overexpression line and its wild-type Zhonghua11.** (a) Grain shape. Scale bar, 3 mm. ZH11, *japonica* cultivar Zhonghua11; *OsOFP8*-OE7, a *OsOFP8*-overexpression line derived from ZH11. (b-c) Grain length (b) and grain length-width ratio (c) of ZH11 and *OsOFP8*-OE7. **, significant difference (*P* < 0.01, *t*-test). (d) Scanning electron microscopy of the outer surfaces of the glumes. Scale bar, 50 μm.


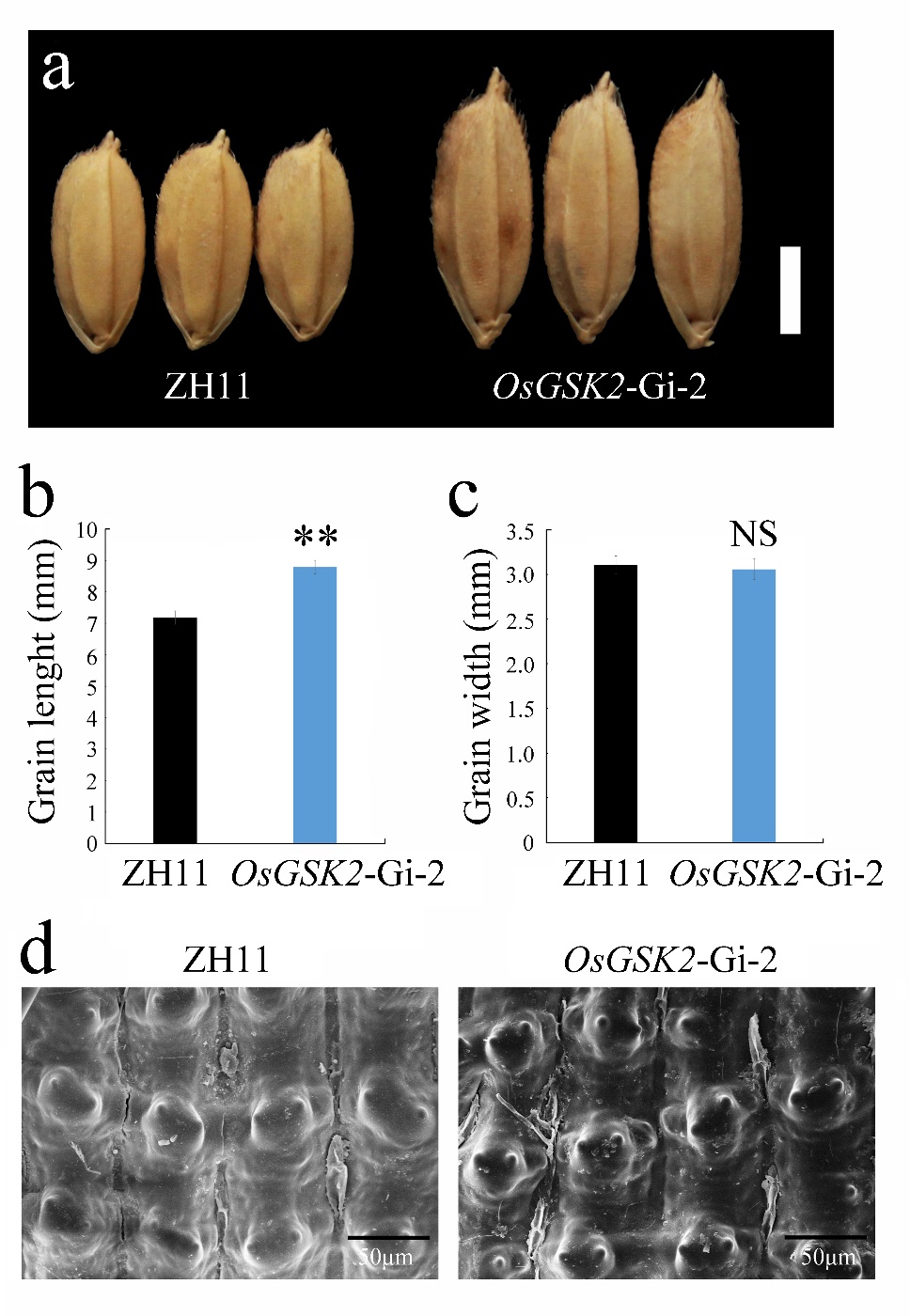


**Supplementary Figure 17.** **Comparison of the mature grains between *OsGSK2*-RNAi line and its wild-type Zhonghua11.** (a) Grain shape. Scale bar, 3 mm. ZH11, *japonica* cultivar Zhonghua11; *OsGSK2*-Gi-2, a *OsGSK2*-RNA interference transgenic line derived from ZH11. (b-c) Grain length (b) and width (c) of ZH11 and *OsGSK2*-Gi-2. **, significant difference (*P* < 0.01, *t*-test); NS, not significant. (d) Scanning electron microscopy of the outer surfaces of the glumes. Scale bar, 50 μm.


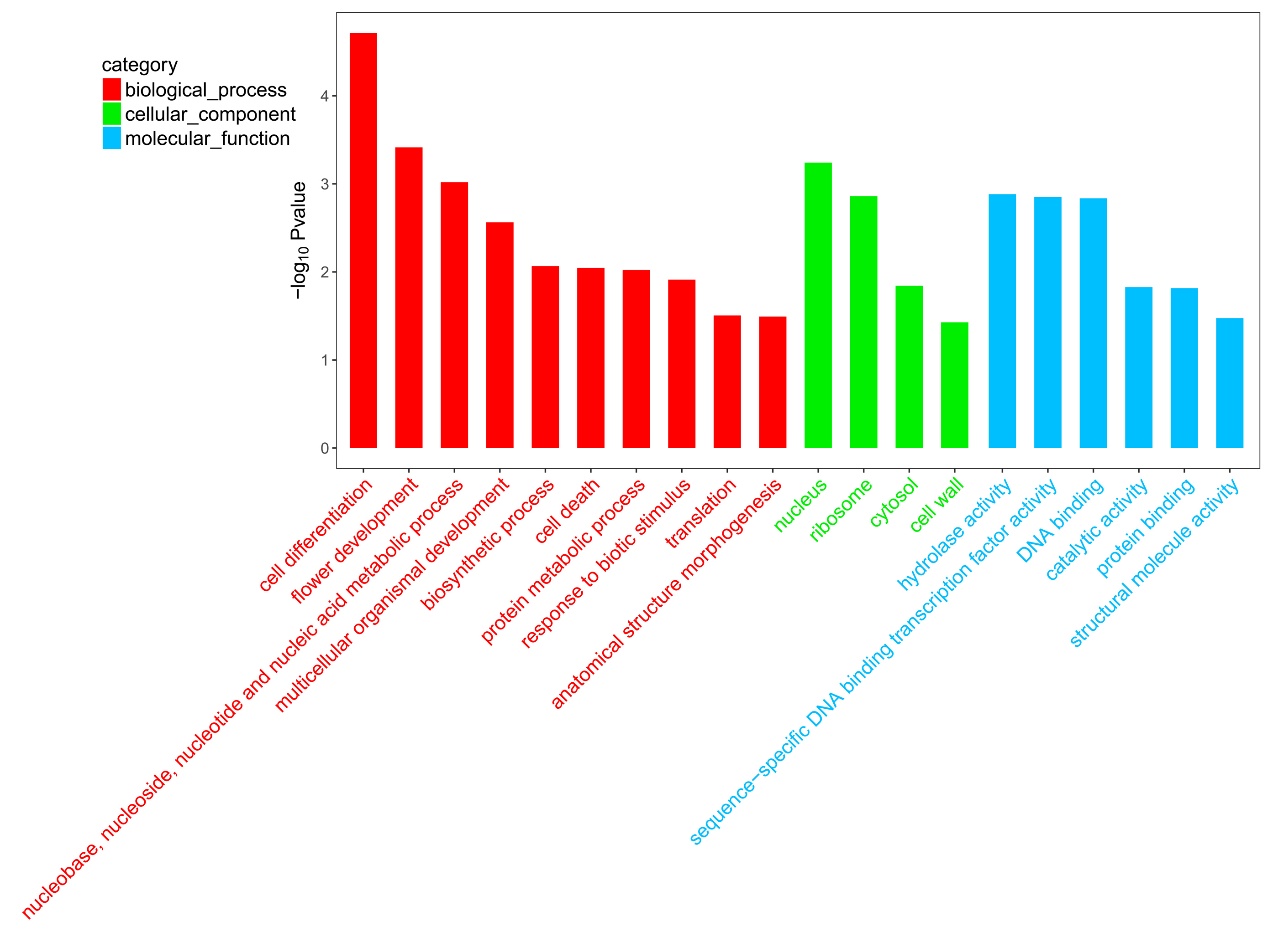


**Supplementary Figure 18.** **GO term analysis of the DEGs from RNA-sequencing data in young panicles between NIL-*gs9* and Nipponbare.** The enriched GO terms of Molecular Function, Biological Process, Cellular Component were listed (*P* < 0.05), and ranked from large to small according to -log_10_Pvalue. Number of corresponding genes of each term should be more than two.


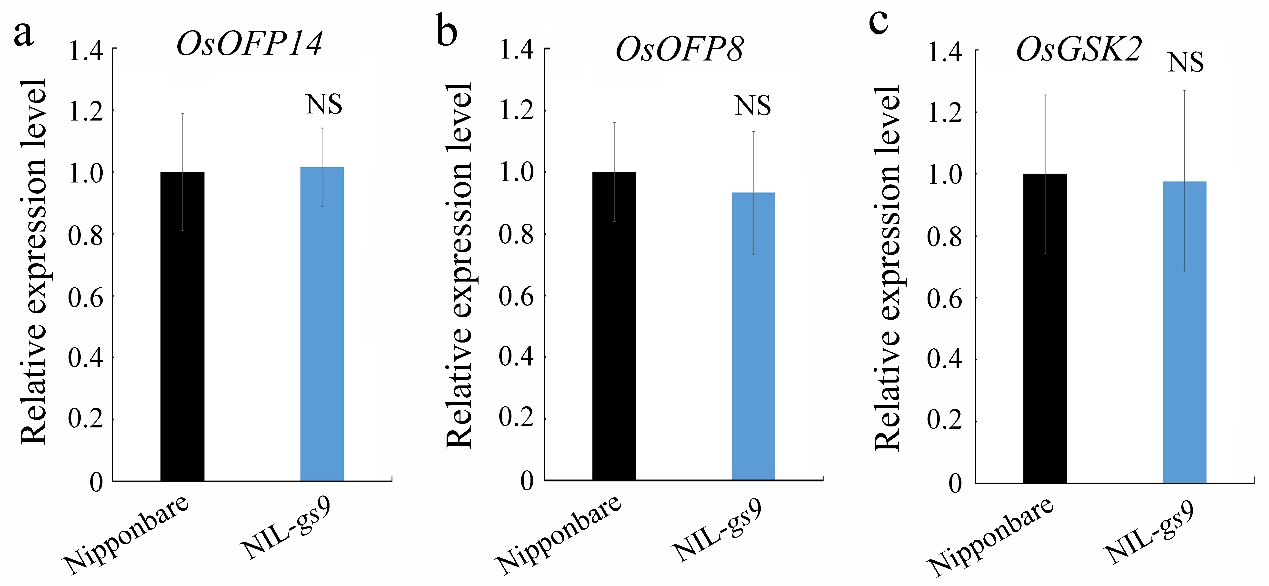


**Supplementary Figure 19.** **Comparison of transcriptional expression levels of GS9 co-regulating related genes in young panicles between NIL-*gs9* and Nipponbare.** (a-c) Transcriptional expression levels of *OsOFP14* (a), *OsOFP8* (b) and *OsGSK2* (c) in young panicles between Nipponbare and NIL-*gs9*. Data are given as means ± SD, with three biological replicates. NS, not significant (*t*-test).


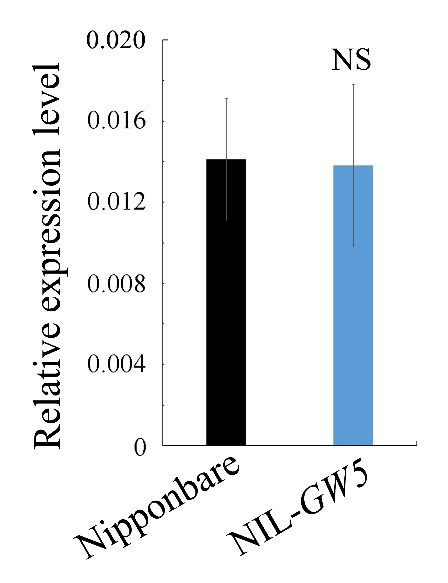


**Supplementary Figure 20.** **Comparison of transcriptional expression level of *GS9* in young panicles between NIL-*GW5* and its recipient Nipponbare (carrying *gw5* allele).** *Actin* was used as the internal control. Data are given as mean ± SD, n=3. NS, not significant difference (*t*-test).


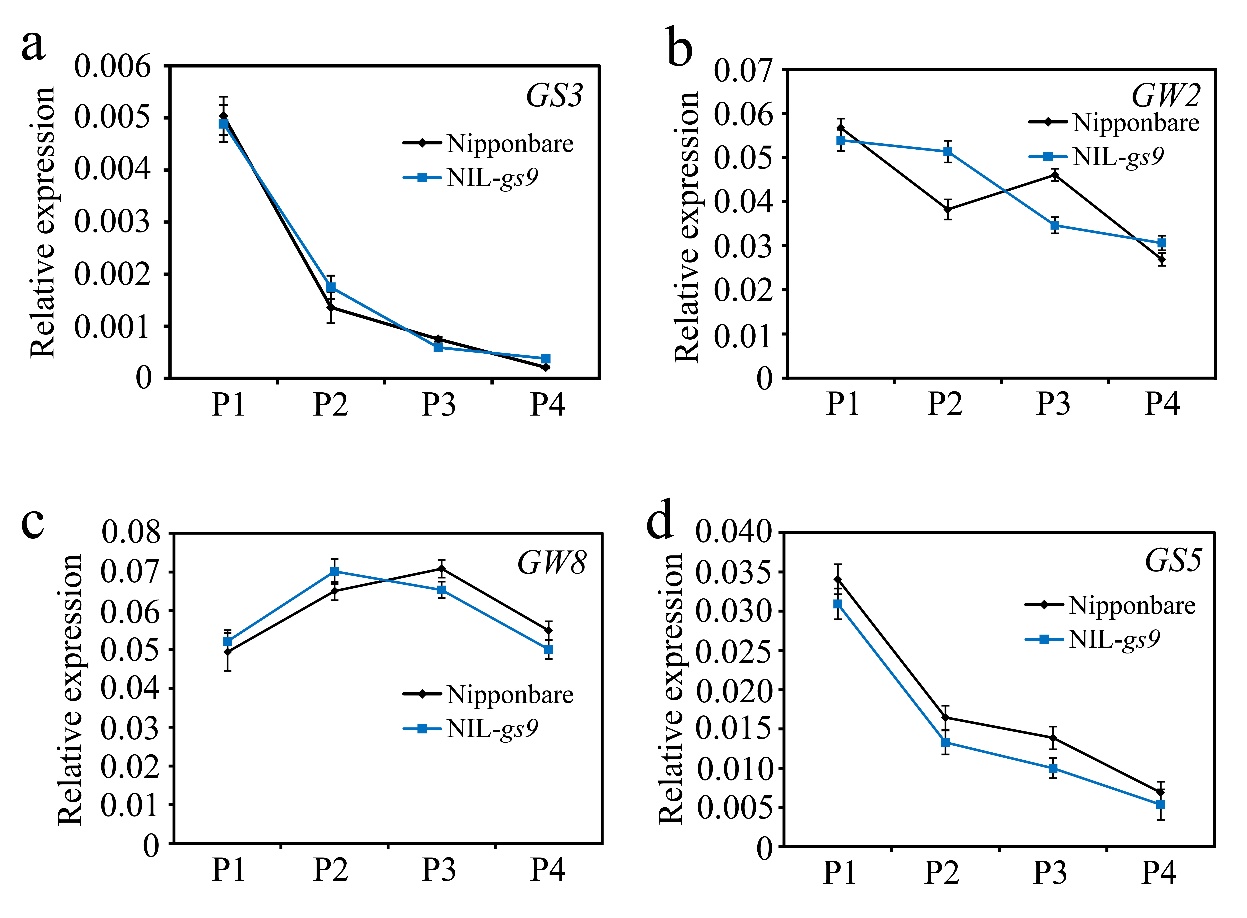


**Supplementary Figure21.** **Comparison of transcriptional expression level of grain size-related genes during panicle development between NIL-*gs9* and its recipient Nipponbare.** (a-d) Transcriptional levels of grain size-related genes *GS3* (a), *GW2* (b), *GW8* (c) and *GS5* (d), respectively. P1~P4 represented the young panicles with length less than 2 cm, 2-5 cm, 5-10 cm, and longer than 10 cm, respectively. *Actin* was used as the internal control. Data are given as means ± SD, n=3.


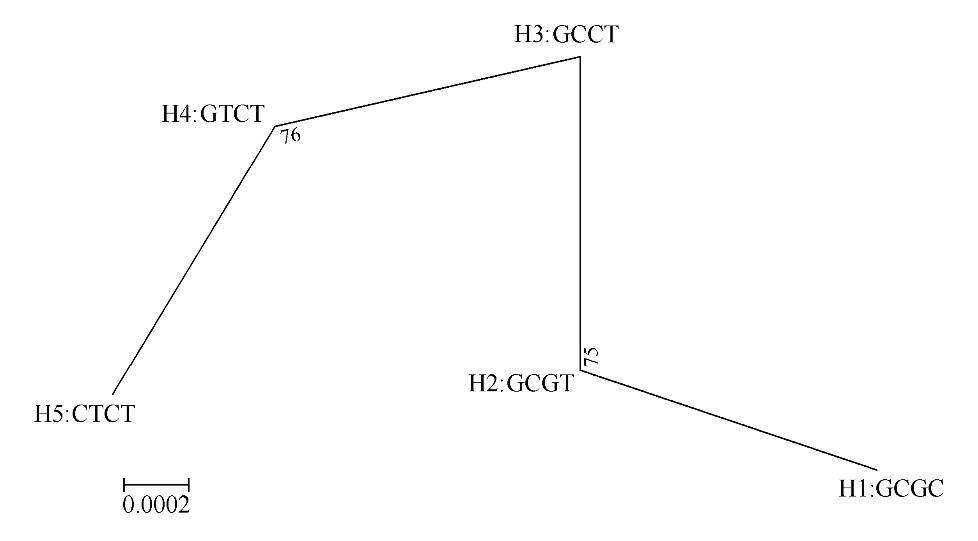


**Supplementary Figure22.** **Phylogenetic tree analysis of five *GS9* haplotypes based on 114 selected rice germplasms.**


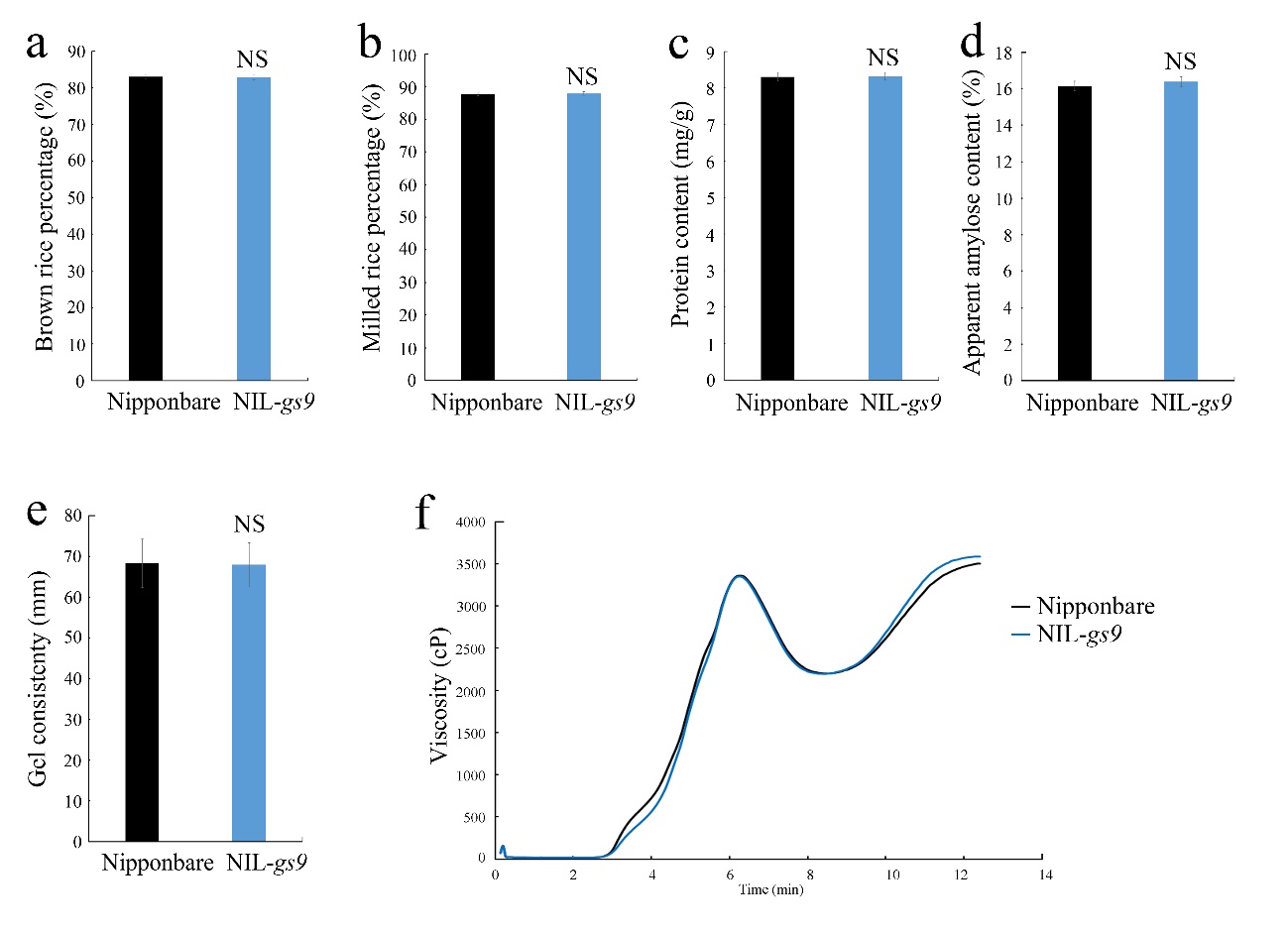


**Supplementary Figure 23.** **Effects of introgression of *gs9* allele on general grain quality in Nipponbare background.** (a-f) Comparison of brown rice percentage (a), milled rice percentage (b), crude protein content (c), apparent amylose content (d), gel consistency (e) and viscosity (f) of mature grains between NIL-*gs9* and its recipient Nipponbare. Data are given as means ± SD, with three biological replicates. NS, not significant difference (*t*-test).


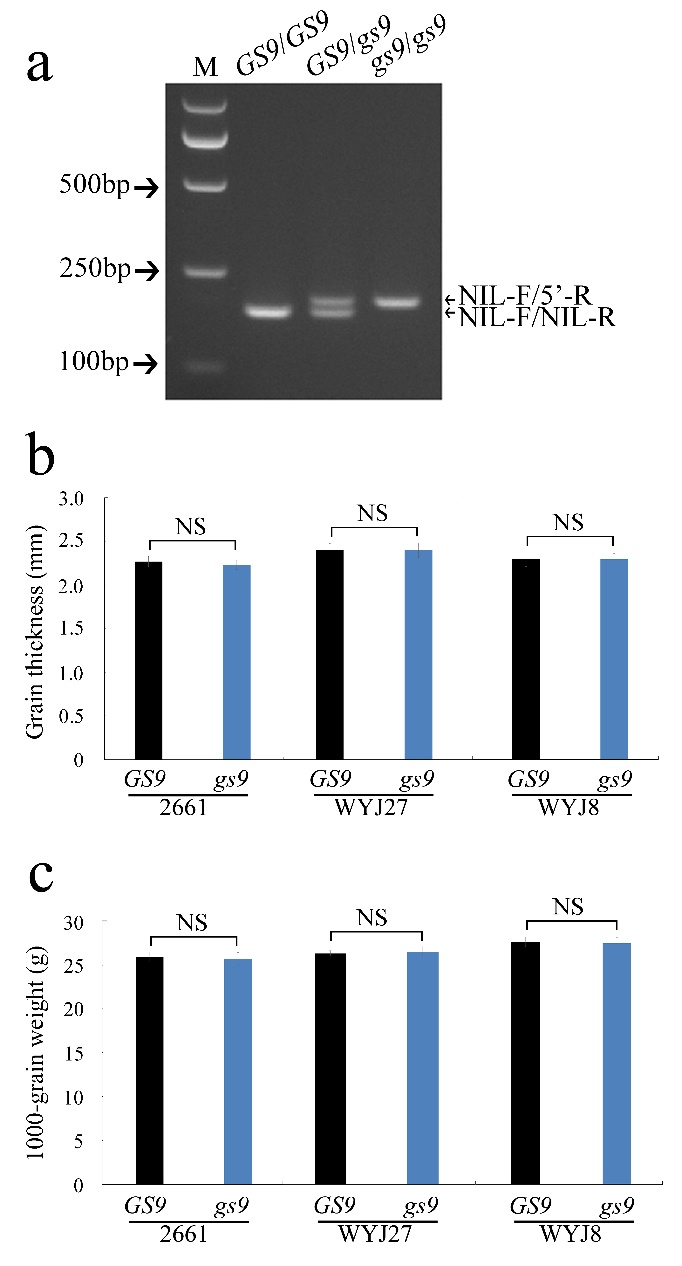


**Supplementary Figure 24**. **Effects of introgression of *gs9* allele on grain shape in released *japonica* cultivars with high yielding.** (a) The three-primer functional marker was designed to detect the *GS9* or *gs9* alleles, which was based on whether the 7-kb insertion in *GS9/gs9*. The location of three primers NIL-F, NIL-R and 5’-R were as shown in Supplementary Figure 3. (b-c) Comparison of grain thickness and 1000-grain weight between the individuals carrying the homozygous *GS9* or *gs9* alleles under *japonica* 2661, WYJ27 or WYJ8 background, respectively. Data are given as means ± SD, n=20 in (b). The individuals carrying the homozygous *GS9* or *gs9* alleles were from the same BC_3_F_2_ population. Data are given as means ± SD, n=3 in (c). NS, not significant level (*t*-test).


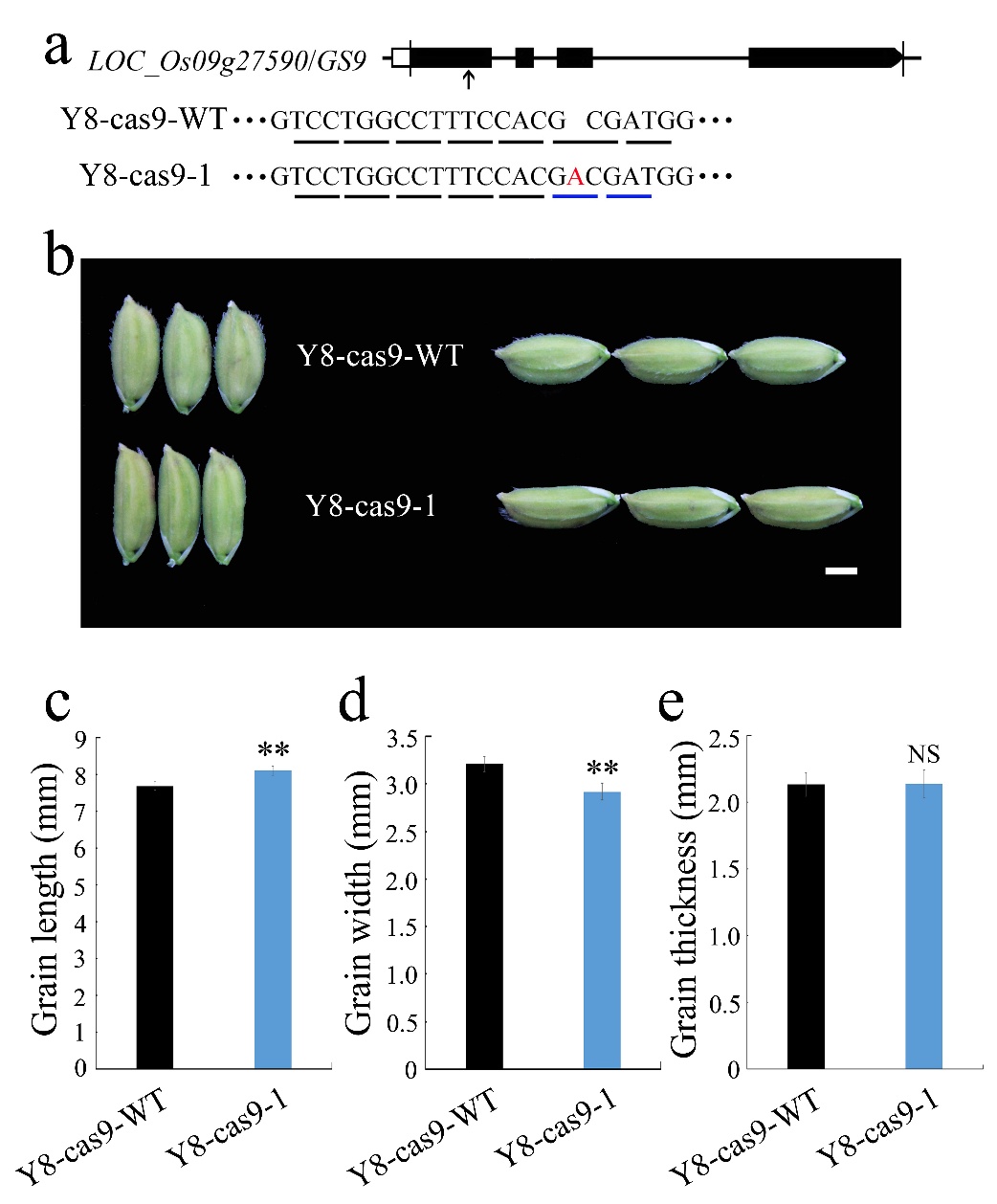


**Supplementary Figure 25.** **The null *gs9* mutant created by CRISPR/Cas9 genome editing system in high-yield *japonica* cultivar Yandao8.** (a) The edited target sequences on *GS9* gene. The vertical arrow indicates the site for editing. Y8-cas9-1, the null *gs9* mutant resulted from a single base insertion into the *GS9* gene of Yangdao8 (Y8). Y8-cas9-WT, non-mutated control. (b-e) Comparison of the morphology of grains (b) and grain shape (c-e) between Y8-cas9-1 and non-mutated plant Y8-cas9-WT. Data are given as means ± SD, n=15. ** means significant difference (*P* < 0.01, *t*-test). NS, not significant level (*t*-test). Scale bar, 2 mm.

**Supplementary Table 1.** Primers of molecular markers used for gene mapping.

| Marker | Physical position | Motif | Primer sequences |
| --- | --- | --- | --- |
| Chr905 | 12,207,935-12,208,122 | Indel | F:TATTTTTCCTGGTGTTAGATCCC |
|  |  |  | R:CTGAGTTTAAATCGGAGTTGTGA |
| RM566 | 14,705,765-14,705,907 | Indel | F:AATATGGTGGCGCGTACATCC |
|  |  |  | R:TGATCGAGCCAACAACAACTGG |
| RI04946 | 15,077,092-15,077,611 | Indel | F:GTCACCATGGACCCCAAC |
|  |  |  | R:GCCTTACCGTAGCAGTACCAG |
| RM3700 | 15,428,388-15,428,282 | Indel | F:AAATGCCCCATGCACAAC |
|  |  |  | R:TTGTCAGATTGTCACCAGGG |
| IN0915 | 16,670,808-16,671,063 | Indel | F:AAGGTATTTGGGCGTGTT |
|  |  |  | R:GCCAGTGTTGCTTACGG |
| RM6235 | 16,675,438-16,675,513 | Indel | F:CGAGACGCAACGTTAGGAGAGC |
|  |  |  | R:CTTCCTCTCATCGATCGAACACC |
| IN0914 | 16,675,431-16,675,545 | Indel | F:ACGCAACCGAGACGCAACG |
|  |  |  | R:GGAGCACAGCACCCTAAACCC |
| IN0916 | 16,710,949-16,711,270 | Indel | F:TCACCCACTCTATCACCATCA |
|  |  |  | R:TGCGATTCTGCCCGTGT |
| IN0917 | 16,731,189-16,731,354 | Indel | F:GTCCCAATGGTGAAACG |
|  |  |  | R:AGCACCGAGACATAAACAA |
| IN0918 | 16,737,116-16,737,270 | Indel | F:GGTTCTTGTGCCTAATCAT |
|  |  |  | R:AACCAGTTACAGCACGAA |
| IN0919 | 16,748,418-16,748,610 | Indel | F:CGTTTAGGCTGGCTGC |
|  |  |  | R:CAGTTGGTGGTTTCGTAGAG |
| IN0921 | 16,754,183-16,754,348 | Indel | F:GGGATCGAGATACTATGGCTGTG |
|  |  |  | R:CCAATCCCGCTACCCTAAT |
| IN0922 | 16,754,379-16,754,472 | Caps | F:GGATCGGGACAATAATATGAAACG |
|  |  |  | R:AACGAGGCTCTGGCTCACG |
| IN0923 | 16,760,267-16,760,449 | Caps | F:GGCAAGCCACATTCGTCT |
|  |  |  | R:AACTGTGATGGGATGATGGC |
| IN0924 | 16,762,784-16,762,890 | Indel | F:AGCCATACACTACAGCAGTTTCA |
|  |  |  | R:GGTTCTATCCGAGTTGGTGTTT |
| IN0925 | 16,763,428-16,763,690 | Caps | F:ATCTGCTCGCCTGTAACTCTTT |
|  |  |  | R:TCCTGGTTCAGAGCGACATT |
| IN0926 | 16,763,598-16,763,849 | Caps | F:AAATGGTAACTTCAGGTCCGATAA |
|  |  |  | R:AAGGTGGCGTTGCCGATG |
| IN0927 | 16,763,671-16,763,849 | Caps | F:AATGTCGCTCTGAACCAGGATG |
|  |  |  | R:AAGGTGGCGTTGCCGATG |
| IN0928 | 16,765,125-16,765,408 | Caps | F:GTCCAAGAGCCGCACGAGCCGATGA |
|  |  |  | R:GGTAGGCGTGGCAAGGATTGGAACTGG |
| IN0930 | 16,767,440-16,767,766 | Caps | F:ACAGAGCAGCAGCAAGCGGCGAA |
|  |  |  | R:TAGCCTCTGGTTCGTATGATGGTGATGGTG |
| IN0929 | 16,769,798-16,769,916 | Caps | F:TCAGTGGCGTGGAGAAC |
|  |  |  | R:TTCAGCAAGAAAGACCAAT |
| IN0911 | 16,774,528-16,774,641 | Indel | F:GGCTGACACTCTGGAACTGGA |
|  |  |  | R:GCCTGGGCAGAGGGTGAG |
| IN0912 | 16,779,574-16,779,669 | Indel | F:CTTTCACAATAGAGGACGAT |
|  |  |  | R:ATTTGTTCCATTCCCATAC |
| IN0920 | 16,827,707-16,827,878 | Indel | F:TTAGAAAAATATTATTATGGGGA |
|  |  |  | R:ATTTAATCTCTTATTTTTTGTCAC |
| IN0913 | 16,851,096-16,851,301 | Indel | F:TCAGCCACCATCACCAAGC |
|  |  |  | R:CGAGCCTGTTGAAGATGACC |
| IN0909 | 16,936,198-16,936,215 | Indel | F:TTTCCGAGGACTTCACTG |
|  |  |  | R:GCTTCAAACCCAACCC |
| IN0906 | 17,287,534-17,287,657 | Indel | F:AAAGCGATACAGGGAAGTCA |
|  |  |  | R:TCGGCTCAGGAGAACAGGA |
| Chr902 | 17,457,769-17,457,915 | Indel | F:TATTAGTAGCGTTTTCTCATTGTGC |
|  |  |  | R:CTGTGGTCTAATCAAACGGTCAT |

**Supplementary Table 2.** Identification of putative GS9-interacting proteins using yeast two-hybrid screening.

| Gene ID | Gene product |
| --- | --- |
| LOC_Os01g57150 | SR protein-related family member |
| LOC_Os02g01740 | DExH-box ATP-dependent RNA helicase DExH12 |
| LOC_Os02g56130 | Proliferating cell nuclear antigen (PCNA) (Cyclin) |
| LOC_Os04g33870 | Transcription repressor OFP14 |
| LOC_Os06g17440 | Microtubule-associated protein |
| LOC_Os08g16780 | DNA-directed RNA polymerase 3 RPC5 |
| LOC_Os09g32440 | Endonuclease / exonuclease / phosphatase family domain |
| LOC_Os09g39750 | DUF966 family protein |
| LOC_Os11g04950 | Splicing factor |
| LOC_Os11g17290 | Ulp1 protease family, C-terminal catalytic domain-containing protein |
| LOC_Os12g16130 | Transposon protein |

The C-terminal truncate of GS9 (GS9-C1) was used as bait as it did not show transcription activation activity. And a prey library was subsequently constructed using total mRNA from young panicles at the early (P1) stage of Nipponbare, when *GS9* expression was highest.

**Supplementary Table 3.** Sequence variation and distribution of *GS9* haplotypes among 114 rice germplasms.

| Haplotype* | InDel1 +154 | SNP1 +1397 | SNP2 +1492 | SNP3 +1521 | SNP4 +1545 | Wild rice** | *Oryza sativa*** | | | | | |
| --- | --- | --- | --- | --- | --- | --- | --- | --- | --- | --- | --- | --- |
|  |  |  |  |  |  |  | *Japonica AROMATIC* | *Japonica TRJ* | *Japonica TEJ* | *Indica AUS* | *Indica IND* | *ADMIX* |
| H1 | — | G | C | G | C |  | 3 | 13 | 18 | 5 |  | 14 |
| H2 | — | G | C | G | T | 22 | 5 |  |  |  |  |  |
| H3 | — | G | C | C | T | 3 |  |  |  |  |  |  |
| H4 | — | G | T | C | T | 1 |  |  |  | 2 | 4 | 1 |
| H5 | GCG | C | T | C | T | 5 |  |  |  |  | 14 | 4 |

TRJ, *tropical japonica*; TEJ, *temperature japonica*; IND, *indica*; ADMIX, admixed accessions.

* The *GS9* gene could be divided into five haplotypes based on identified sequence variation.

** Numbers indicate the amount of each haplotype within certain population.

**Supplementary Table 4.** The *GS9* haplotypes among 31 wild rice in this study.

| *GS9* haplotype* | Accession No. | Sub-population |
| --- | --- | --- |
| H2 | A102 | *Oryza rufipogon* |
| H2 | A201 | *Oryza glaberrima* |
| H2 | A202 | *Oryza glaberrima* |
| H2 | A203 | *Oryza glaberrima* |
| H2 | A204 | *Oryza glaberrima* |
| H2 | A301 | *Oryza nivara* |
| H2 | A303 | *Oryza nivara* |
| H2 | A402 | *Oryza glumaepatula* |
| H2 | A404 | *Oryza glumaepatula* |
| H2 | ZCW-17 | *Oryza rufipogon* |
| H2 | ZCW-22 | *Oryza rufipogon* |
| H2 | ZCW-68 | *Oryza rufipogon* |
| H2 | ZCW-90 | *Oryza rufipogon* |
| H2 | GZW-38 | *Oryza rufipogon* |
| H2 | GZW-39 | *Oryza rufipogon* |
| H2 | GZW-40 | *Oryza rufipogon* |
| H2 | GZW-44 | *Oryza rufipogon* |
| H2 | GZW-110 | *Oryza rufipogon* |
| H2 | GZW-112 | *Oryza rufipogon* |
| H2 | GZW-125 | *Oryza rufipogon* |
| H2 | SXW-10 | *Oryza rufipogon* |
| H2 | SXW-57 | *Oryza rufipogon* |
| H3 | A101 | *Oryza rufipogon* |
| H3 | A103 | *Oryza rufipogon* |
| H3 | A104 | *Oryza rufipogon* |
| H4 | A302 | *Oryza nivara* |
| H5 | QHW-67 | *Oryza rufipogon* |
| H5 | QHW-68 | *Oryza rufipogon* |
| H5 | A105 | *Oryza rufipogon* |
| H5 | A401 | *Oryza glumaepatula* |
| H5 | A403 | *Oryza glumaepatula* |

* *GS9* haplotypes are the same as shown in Supplementary Table 5. **Supplementary Table 5.** The *GS9* haplotypes among 83 rice cultivars in this study.

| *GS9* haplotype** | Cultivar name | Country of origin | Sub-population* |
| --- | --- | --- | --- |
| H1 | Sadri Belyi | Azerbaijan | AROMATIC |
| H1 | Basmati | Pakistan | AROMATIC |
| H1 | NSF-TV 160 | Iran | AROMATIC |
| H1 | Arias | India | TRJ |
| H1 | Asse Y Pung | Philippines | TRJ |
| H1 | Caawa/Fortuna 6-103-15 | Taiwan | TRJ |
| H1 | Carolina Gold Sel | United States | TRJ |
| H1 | Iguape Cateto | Haiti | TRJ |
| H1 | NSF-TV 27 | Pakistan | TRJ |
| H1 | Sultani | Egypt | TRJ |
| H1 | Fossa Av | Burkina Faso | TRJ |
| H1 | WC 3397 | Jamaica | TRJ |
| H1 | WC 4443 | Bolivia | TRJ |
| H1 | WAB 502-13-4-1 | Cote D'Ivoire | TRJ |
| H1 | Creole | Belize | TRJ |
| H1 | 318 | Turkey | TRJ |
| H1 | Baber | India | TEJ |
| H1 | Baghlani Nangarhar | Afghanistan | TEJ |
| H1 | Chinese | China | TEJ |
| H1 | Chodongji | South Korea | TEJ |
| H1 | Early Wataribune | Japan | TEJ |
| H1 | Kaw Luyoeng | Thailand | TEJ |
| H1 | Bellardone | France | TEJ |
| H1 | Benllok | Peru | TEJ |
| H1 | Yabani Montakhab 7 | Egypt | TEJ |
| H1 | Desvauxii | Former Soviet Union | TEJ |
| H1 | Vavilovi | Kazakhstan | TEJ |
| H1 | Gambiaka Sebela | Mali | TEJ |
| H1 | Chibica | Mozambique | TEJ |
| H1 | Taipei309 | Taiwan | TEJ |
| H1 | Nipponbare | Japan | TEJ |
| H1 | Yandao8 | China | TEJ |
| H1 | Wuyunjing27 | China | TEJ |
| H1 | Wuyunjing8 | China | TEJ |
| H1 | Mehr | Iran | AUS |
| H1 | CA 902/B/2/1 | Chad | AUS |
| H1 | Saraya | Fiji | AUS |
| H1 | Paung Malaung | Myanmar | AUS |
| H1 | Shirkati | Afghanistan | AUS |
| H1 | ARC 10086 | India | ADMIX |
| H1 | Breviaristata | Portugal | ADMIX |
| H1 | Victoria F.A. | Argentina | ADMIX |
| H1 | Habiganj Boro 6 | Bangladesh | ADMIX |
| H1 | Doble Carolina Rinaldo Barsani | Uruguay | ADMIX |
| H1 | Saku | Mongolia | ADMIX |
| H1 | Pirinae 69 | Former Yugoslavia | ADMIX |
| H1 | Guineandao | Guinea | ADMIX |
| H1 | Baldo | Italy | ADMIX |
| H1 | Tokyo Shino Mochi | Japan | ADMIX |
| H1 | Saturn | United States | ADMIX |
| H1 | Keriting Tingii | Indonesia | ADMIX |
| H1 | KU115 | Thailand | ADMIX |
| H1 | K 65 | Suriname | ADMIX |
| H2 | NSF-TV 5 | India | AROMATIC |
| H2 | Bico Branco | Brazil | AROMATIC |
| H2 | Lambayeque 1 | Peru | AROMATIC |
| H2 | Dom Zard | Iran | AROMATIC |
| H2 | Firooz | Iran | AROMATIC |
| H4 | Kalubala Vee | Sri Lanka | AUS |
| H4 | Kaukau | Mali | AUS |
| H4 | Radin Ebos 33 | Malaysia | IND |
| H4 | Tchibanga | Gabon | IND |
| H4 | Byakkoku Y 5006 Seln | Australia | IND |
| H4 | Radin Ebos 33 | Malaysia | IND |
| H4 | Blue Rose | Louisiana | ADMIX |
| H5 | Chau | Vietnam | IND |
| H5 | ECIA76-S89-1 | Cuba | IND |
| H5 | Djimoron | Guinea | IND |
| H5 | Sundensis | Kazakhstan | IND |
| H5 | LD 24 | Sri Lanka | IND |
| H5 | SML 242 | Suriname | IND |
| H5 | BR24 | Bangladesh | IND |
| H5 | Dawebyan | Myanmar | IND |
| H5 | Teqing | China | IND |
| H5 | Minghui63 | China | IND |
| H5 | IR64 | Philippines | IND |
| H5 | Zhenshan97 | China | IND |
| H5 | Qingluzhan11 | China | IND |
| H5 | 9311 | China | IND |
| H5 | Tsipala 421 | Madagascar | ADMIX |
| H5 | Riz Local | Burkina Faso | ADMIX |
| H5 | Sadri Tor Misri | Iran | ADMIX |
| H5 | C1-6-5-3 | Mexico | ADMIX |

* TRJ, *tropical japonica*; TEJ, *temperature japonica*; IND, *indica*; ADMIX, admixed accessions.

** *GS9* haplotypes are the same as shown in Supplementary Table 5.

**Supplementary Table 6.** Primers used for transcriptional expression analyses, gene sequencing and vector construction.

| Primer | Sequences | Function |
| --- | --- | --- |
| GS3 | F: GAACTCCTGATCCATTCATAACGATT | qRT-PCR expression analysis |
|  | R: CAAACAGCGAAACTTCTTCAAGAA |  |
| GS5 | F: CATTCCATGCAAATGCCAGTGGAC |  |
|  | R: CAGCCCTGCTTTGATGAGCTTG |  |
| GW2 | F: CAGCAGCGCATTCCCAGTTTTC |  |
|  | R: GTGGTCAGCCGAGCACTCTC |  |
| GW8 | F: AGGAGTTTGATGAGGCCAAG |  |
|  | R: GCGTGTAGTATGGGCTCTCC |  |
| GW5 | F: TGGGATATGGAATGGAATGGGTTGG |  |
|  | R: GATAGGGGTGGGGATGGGATGAATG |  |
| RT590N | F: GACGCAAGCAGCGACCAGCA |  |
|  | R: GCCTGGCAGTTGGAGGATGAGC |  |
| RT590C | F: CGCCCGTTCGCGGTGCT |  |
|  | R: CCTCTGGTTCGTATGATGGTGATGG |  |
| seq590p-3 | F: GCTTTCGTTGCTTCATTTGG | Gene sequencing analysis |
|  | R: TGGAAGACCGAGACGAGGC |  |
| seq590p-2 | F: CTCACAAGTCGCAAGTCCC |  |
|  | R: TGTATGATTTTTAGATTTAGAGGTG |  |
| seq590p-1 | F: GCCTCTACAATCACACAAAAATACA |  |
|  | R: TCTGTGTGTGCTGTTGGCG |  |
| seq590m | F: TCATCCTACTCGGCAACTCACTCTG |  |
|  | R: CATCGTCTTCGTCTTCGTCGTCA |  |
| seq590g-1 | F: TCACTCATCCTACTCGGCAACTCA |  |
|  | R: GTCCCATCGCCATTGACAGAA |  |
| seq590g-2 | F: GATTCTGTCAATGGCGATGGG |  |
|  | R: TAGCCTCTGGTTCGTATGATGGTG |  |
| seq590g-3 | F: GGCACCATCACCATCATACGAACC |  |
|  | R: AGGCCCTGAACCAAAGATGAGAATT |  |
| TOPO590 | F: CACCATGGAGGCAGCAGCCCAAGAAA | Vector construction |
|  | R: GCCTCTGGTTCGTATGATGGTGATG |  |
| TOPO-OFP14 | F: CACCATGCCTCCCCTGAACCCTCCCCACC |  |
|  | R: GCTCTCCTCCTCCATGGTCGCCGA |  |
| p1300-590FL | F: GGTACCTCATCCTACTCGGCAACTCACTCTG |  |
|  | R: GAATTCCATCGTCTTCGTCTTCGTCGTCA |  |
| p1300-590RF | F: GGTACCATGCAGAGCAGCAGCAAGCGGC |  |
|  | R: GAATTCCATCGTCTTCGTCTTCGTCGTCA |  |
| p1300-590RF1 | F: GGTACCATGAGGGTGCTGCGCCCGTT |  |
|  | R: GAATTCCATCGTCTTCGTCTTCGTCGTCA |  |
| p1300-590NRF | F: GGTACCTCATCCTACTCGGCAACTCACTCTG |  |
|  | R: GAATTCCATCGTCTTCGTCTTCGTCGTCA |  |
| p1300-590pro | F: AAGCTTTGTCGCTCTGAACCAGGATG |  |
|  | R: GGTACCGCCGAGTAGGATGAGTGAGGA |  |
| p1301-590pro | F: AAGCTTTGTCGCTCTGAACCAGGATG |  |
|  | R: CCATGGGCCGAGTAGGATGAGTGAGGA |  |
| cas590 | F: GTCCTGGCCTTTCCACGCGA |  |
| 590BD | F: CATATGTCATCCTACTCGGCAACTCACTCTG |  |
|  | R: GTCGACCATCGTCTTCGTCTTCGTCGTCA |  |
| 590BD-NRF | F: CATATGTCATCCTACTCGGCAACTCACTCTG |  |
|  | R: GTCGACTCGCCGCTTGCTGCTGCTCT |  |
| 590BD-RF | F: CATATGATGCAGAGCAGCAGCAAGCGGC |  |
|  | R: GTCGACCATCGTCTTCGTCTTCGTCGTCA |  |
| 590BD-RF1 | F: CATATGATGAGGGTGCTGCGCCCGTT |  |
|  | R: GTCGACCATCGTCTTCGTCTTCGTCGTCA |  |
| OFP14AD | F: GAATTCGCAATGCCTCCCCTGAACCC |  |
|  | R: GGATCCTCCCTCACTTGCCATTCCTTCGT |  |
| OFP1AD | F: GAATTCTACTGGTGGTAGCTGCCAATGG |  |
|  | R: GGATCCGGAGTAGTACTCTGGCTAATCTCAGTGAT |  |
| OFP8AD | F: GAATTCCCAGTGGCATTGTCCATGTCG |  |
|  | R: GGATCCGGCGTCGCGTTGAAACTTGG |  |
| GSK2AD | F: CATATGAGCTTCCAGCTTTGCCACAT |  |
|  | R: GAATTCAGAATCATCTGACTCAGCCTTCAT |  |
| OFP14BD | F: GAATTCGCAATGCCTCCCCTGAACCC |  |
|  | R: GGATCCTCCCTCACTTGCCATTCCTTCGT |  |
| GSK2BD | F: CATATGAGCTTCCAGCTTTGCCACAT |  |
|  | R: GAATTCAGAATCATCTGACTCAGCCTTCAT |  |
| 590AD-RF | F: CATATGATGCAGAGCAGCAGCAAGCGGC |  |
|  | R: GAATTCCATCGTCTTCGTCTTCGTCGTCA |  |
| p2300-590 | F: GGTACCTCATCCTACTCGGCAACTCACTCTG |  |
|  | R: GTCGACGCCTCTGGTTCGTATGATGGTGAT |  |
| p2300-OFP14 | F: GGATCCGCAATGCCTCCCCTGAACCC |  |
|  | R: GAATTCTCCCTCACTTGCCATTCCTTCGT |  |
| P163-GS9GFP | F: AAGCTTTCATCCTACTCGGCAACTCACTCTG |  |
|  | R: GTCGACGCCTCTGGTTCGTATGATGGTGAT |  |
| P163-OFP14GFP | F: AAGCTTGCAATGCCTCCCCTGAACCC |  |
|  | R: GGATCCTCCCTCACTTGCCATTCCTTCGT |  |
| pYNE-GS9 | F: GTCGACTCATCCTACTCGGCAACTCACTCTG |  |
|  | R: GGTACCCATCGTCTTCGTCTTCGTCGTCA |  |
| pYCE-OFP14 | F: GGATCCGCAATGCCTCCCCTGAACCC |  |
|  | R: CCCGGGTCCCTCACTTGCCATTCCTTCGT |  |
| pYCE-OFP8 | F: GGATCCCCAGTGGCATTGTCCATGTCG |  |
|  | R: GGTACCGGCGTCGCGTTGAAACTTGG |  |
| pMN6-590 | F: ACTAGTTCATCCTACTCGGCAACTCACTCTG |  |
|  | R: GGTACCCATCGTCTTCGTCTTCGTCGTCA |  |
| pMN6-OFP8 | F: ACTAGTCCAGTGGCATTGTCCATGTCG |  |
|  | R: GGTACCGGCGTCGCGTTGAAACTTGG |  |
| pJIT-OFP14 | F: AAGCTTGCAATGCCTCCCCTGAACCC |  |
|  | R: GGATCCTCCCTCACTTGCCATTCCTTCGT |  |
| pJIT-OFP8 | F: AAGCTTCCAGTGGCATTGTCCATGTCG |  |
|  | R: GGATCCGGCGTCGCGTTGAAACTTGG |  |
| pJIT-GSK2 | F: GTCGACAGCTTCCAGCTTTGCCACAT |  |
|  | R: GTCGACAGAATCATCTGACTCAGCCTTCAT |  |
