# Supplementary material for: GS9 acts as a transcriptional activator to regulate rice grain shape and appearance quality
Source: Nat Commun. 2018 Mar 27;9:1240. doi: 10.1038/s41467-018-03616-y (PMC5869696; doi:10.1038/s41467-018-03616-y)
Supplement: Supplementary file 2 — Descriptions of Additional Supplementary Files(PDF 25 kb) [file 41467_2018_3616_MOESM2_ESM.pdf]

Descriptions of Additional Supplementary Files

File name: Supplementary Data 1
Description: A list of global differentially expressed genes (DEGs) in young panicles between NIL-*gs9* (*gs9*) and Nipponbare (NPB). The database contains a list of significantly ($FDR < 0.05$) up- or down-regulated genes with 2-fold or 1/2-fold change, respectively, in NIL-*gs9* (*gs9*) compared to Nipponbare (NPB).

File name: Supplementary Data 2
Description: Comparison of transcript abundance of rice *OFP* genes, and genes involved in BR signal pathway in young panicles between NIL-*gs9* (*gs9*) and Nipponbare (NPB). All the data were from the RNA-sequencing analyses as mentioned in Methods.
